# A systematic review of cerebral microdialysis and outcomes in TBI: relationships to patient functional outcome, neurophysiologic measures, and tissue outcome

**DOI:** 10.1007/s00701-017-3338-2

**Published:** 2017-10-07

**Authors:** Frederick A. Zeiler, Eric Peter Thelin, Adel Helmy, Marek Czosnyka, Peter J. A. Hutchinson, David K. Menon

**Affiliations:** 1 Section of Neurosurgery, Department of Surgery, Rady Faculty of Health Sciences, University of Manitoba, Winnipeg, MB R3A 1R9 Canada; 2 Clinician Investigator Program, University of Manitoba, Winnipeg, Canada; 3 Department of Anesthesia, Addenbrooke’s Hospital, University of Cambridge, Cambridge, UK; 4 Division of Neurosurgery, Department of Clinical Neurosciences, University of Cambridge, Cambridge Biomedical Campus, Cambridge, CB2 0QQ UK; 5 Department of Clinical Neuroscience, Neurosurgical Research Laboratory, Karolinska University Hospital, Building R2:02, Karolinska Institutet, S-17176 Stockholm, Sweden; 6 Section of Brain Physics, Division of Neurosurgery, University of Cambridge, Cambridge, CB2 0QQ UK; 7 Neurosciences Critical Care Unit, Addenbrooke’s Hospital, Cambridge, UK; 8 Queens’ College, Cambridge, UK; 9 National Institute for Health Research, Southampton, UK

**Keywords:** Cerebral microdialysis, Systematic review, Patient outcome, Functional outcome

## Abstract

**Objective:**

To perform a systematic review on commonly measured cerebral microdialysis (CMD) analytes and their association to: (A) patient functional outcome, (B) neurophysiologic measures, and (C) tissue outcome; after moderate/severe TBI. The aim was to provide a foundation for next-generation CMD studies and build on existing pragmatic expert guidelines for CMD.

**Methods:**

We searched MEDLINE, BIOSIS, EMBASE, Global Health, Scopus, Cochrane Library (inception to October 2016). Strength of evidence was adjudicated using GRADE.

**Results:**

(A) *Functional Outcome*: 55 articles were included, assessing outcome as mortality or Glasgow Outcome Scale (GOS) at 3–6 months post-injury. Overall, there is GRADE C evidence to support an association between CMD glucose, glutamate, glycerol, lactate, and LPR to patient outcome at 3–6 months. (B) *Neurophysiologic Measures*: 59 articles were included. Overall, there currently exists GRADE C level of evidence supporting an association between elevated CMD measured mean LPR, glutamate and glycerol with elevated ICP and/or decreased CPP. In addition, there currently exists GRADE C evidence to support an association between elevated mean lactate:pyruvate ratio (LPR) and low PbtO_2_. Remaining CMD measures and physiologic outcomes displayed GRADE D or no evidence to support a relationship. (C) *Tissue Outcome*: four studies were included. Given the conflicting literature, the only conclusion that can be drawn is acute/subacute phase elevation of CMD measured LPR is associated with frontal lobe atrophy at 6 months.

**Conclusions:**

This systematic review replicates previously documented relationships between CMD and various outcome, which have driven clinical application of the technique. Evidence assessments do not address the application of CMD for exploring pathophysiology or titrating therapy in individual patients, and do not account for the modulatory effect of therapy on outcome, triggered at different CMD thresholds in individual centers. Our findings support clinical application of CMD and refinement of existing guidelines.

**Electronic supplementary material:**

The online version of this article (10.1007/s00701-017-3338-2) contains supplementary material, which is available to authorized users.

## Introduction

Cerebral microdialysis (CMD) has seen broad application across neurocritical care [19, 24, 46]. By sampling brain parenchymal extracellular fluid, CMD provides insights into cellular metabolism and injury to guide clinical therapy, and provide characterization of the host response (currently only as a research tool) during pathologic states. To date, the largest application of CMD is within the traumatic brain injury (TBI) population [19, 46].

Commonly measured CMD analytes include: glucose, lactate, pyruvate, (allowing calculation of the lactate:pyruvate ratio (LPR)), glutamate, glycerol as well as the cations sodium and potassium [41, 49, 50] (although the common presence of these cations in the dialysis fluid makes their measurement challenging). Other potential candidate biomarkers can be measured from CMD samples, and an expanding panel includes immune cytokines and other biomolecules [35, 36]. However, these latter analytes are measured offline and do not currently impact clinical practice.

Despite the wide application of CMD in TBI in neuro-intensive care units (NICUs), the technique is still developing, and the evidence base supporting its optimal use evolving. Published expert consensus statements on the application of multi-modal intracranial monitoring in general, and microdialysis in particular, support the use of CMD as part of a multi-modality panel [41, 49, 50]. These consensus statements summarize key publications and provide a pragmatic approach to the use of CMD based on collective current experience. Critically, much of this advice has been based on outcome associations of monitored variables. However, there has been no detailed systematic review of the literature using standard evidence evaluation methodology.

The absence of such a review is unsurprising for several reasons. First, the studies are heterogenous, and with a few exceptions, involve small patient numbers. Second, intervention thresholds based on microdialysis monitoring in these studies vary substantially, and the consensus achieved in recent publications has not been universally applied in the past. Finally, despite the outcome associations that have been demonstrated, any impact of a monitoring technique on outcome will critically depend on the interventions that it triggers, and these are both highly variable between centers, and generally poorly described in the available literature. Consolidation of the evidence base for optimal use of CMD may require a new generation of studies, which couples the best estimate of critical monitoring thresholds identified by expert opinion and physiological principles with standard approaches to data collection and outcome assessment.

Critically, this new generation of studies will need a framework for protocols that complies with quality metrics that underpin evidence-based medicine. The development of such a framework is dependent on a robust review of the available literature, not just summarizing the evidence, but also identifying whether and how a subsequent generation of studies can be designed to provide further high-quality evidence to underpin clinical practice. We have therefore undertaken a rigorous systematic review of the available literature addressing three key questions:

- Do commonly measured CMD analytes display an association with patient functional outcome?
- Are these analytes associated with specific neurophysiologic changes seen during NICU care?
- Are these analytes associated with tissue outcome on imaging (such as acute ischemia/infarct, or atrophy long term)?

## Methods

A systematic review was conducted using the methodology outlined in the Cochrane Handbook for Systematic Reviewers [37]. Data were reported following the Preferred Reporting Items for Systematic Reviews and Meta-Analyses (PRISMA) [59]. The review questions and search strategy were decided upon by the primary author (FAZ) and supervisors (AH and DKM).

### Search question, population, inclusion and exclusion criteria

The questions posed for this systematic review were:

1. Do commonly measured CMD analytes have an association with patient functional outcome in moderate and severe TBI?
2. Do commonly measured CMD analytes have an association with neuro-physiologic measures in moderate and severe TBI?
3. Do commonly measured CMD analytes have an association with tissue outcome/tissue fate in moderate and severe TBI?

The CMD analytes that we included in the “commonly measured” category included: glucose, lactate, pyruvate, LPR, potassium, sodium, glutamate, and glycerol. All studies of five patients or more were included.

#### Functional outcome – outcome measures and primary/secondary outcomes

The primary outcome measure was: documented association between one of the CMD measures and patient outcome. Any outcome score or mention of morbidity/mortality within the studies was deemed acceptable. Secondary outcome measures were: complications associated with CMD monitoring.

#### Neuro-physiologic measures – outcome measures and primary/secondary outcomes

The physiologic parameters of interest were: ICP, CPP, PbtO2, cerebral physiology and metabolism assessed using PET, autoregulatory capacity (measured by any technique), and CBF (assessed by any technique). All studies of five patients or more were included. The primary outcome measure was: documented association between one of the CMD measures and one of the mentioned neuro-physiologic measures. Secondary outcome measures were: complications associated with CMD monitoring.

#### Tissue outcome – outcome measures and primary/secondary outcomes

The tissue outcome of interest was ischemia/infarction during acute/subacute hospital stay, primary lesion expansion during acute/subacute stay, and cortical atrophy as assessed during long-term follow-up imaging. Imaging studies deemed appropriate endpoints for association with CMD variables were computed tomography (CT), magnetic resonance imaging (MRI)/magnetic resonance spectroscopy (MRS), and a subset of positron emission tomography (PET) studies (see below). All studies of five patients or more were included. The primary outcome measure was: documented association between one of the CMD measures and tissue outcome. Secondary outcome measures were: complications associated with CMD monitoring.

#### General inclusion/exclusion criteria

Inclusion criteria were: All studies including human subjects with moderate or severe TBI (GCS of 12 or less), studies with five or more patients, any age category, the use of CMD to measure “common” analytes (glucose, lactate, pyruvate, LPR, glutamate, glycerol) as well as potassium and sodium, and documentation of either: (A) patient functional outcome in relation to CMD measures, (B) documentation of neuro-physiologic metrics in relation to CMD analytes, (C) the use CMD to measure “common” analytes (glucose, lactate, pyruvate, LPR, glutamate, glycerol), and documentation of tissue outcome in relation to CMD measures.

Exclusion criteria were: Non-English studies, animal studies and studies of less than five patients. Non-English studies were excluded given the small number identified. For the “tissue outcome” portion of the review, positron emission tomography (PET) tissue assessments based on ^15^O PET or [^18^F] FDG PET were excluded due to the fact that these imaging modalities assess neurophysiologic parameters such as metabolism and cerebral blood flow (CBF), not tissue outcomes such as ischemia/infarction or cortical volume loss. However, we sought to include PET studies that related CMD variables to PET ligands that mapped survival of neuronal populations at follow up (e.g., [^11^C]flumazenil PET and GaBAergic neurons).

### Search strategy

MEDLINE, BIOSIS, EMBASE, Global Health, SCOPUS, and Cochrane Library from inception to October 2016 were searched using individualized search strategies. The search strategy for MEDLINE can be seen in Appendix A of the supplementary material, with a similar search strategy utilized for the other databases.

In addition, we surveyed relevant meeting proceedings for the last 5 years for ongoing and unpublished work based on CMD in moderate-to-severe TBI patients. The meeting proceedings of the professional societies that were searched can be seen in Appendix A of the Supplementary materials.

Finally, reference lists of any review articles on CMD were searched for relevant studies on CMD.

### Study selection

Two reviewers undertook a two-step review of all articles returned by our search strategies, separately for each review question resulting in three separate systematic reviews.

First, reviewers independently (FZ and ET) screened titles and abstracts of the returned articles to decide if they met inclusion criteria. Second, full text of the chosen articles were then assessed to confirm if they met the inclusion criteria and that the primary outcome of patient functional outcome was reported in the study (FZ and ET). Any discrepancies between the two reviewers were resolved by a third reviewer if needed (AH or DKM).

### Data collection

Data were extracted from the selected articles and stored in an electronic database. Data fields included: patient demographics, type of study, article location, number of patients, CMD analyte measured, CMD measurement details (probe tissue location, sampling frequency), outcome measure utilized (outcome score, neuro-physiologic variable measured, tissue outcome assessment technique etc.), association of CMD measure to outcome (functional, neuro-physiologic, or tissue), and complications to CMD.

### Bias assessment

Two reviewers (FZ and RT) used the RTI item bank [102] to assess bias in each study, with each component item graded as Low-risk, High-risk, or Unclear. Discrepancies resolved by discussion and a third party if needed (AH or DKM). This bias assessment was repeated for each question of interest, as there was some overlap in the articles between the review on patient functional outcome and neuro-physiologic measures. Appendix B in the Supplementary Material provides a table outlining details of risk assessment.

### Quality of evidence assessment

Each reviewer (FZ and ET) used the Grading of Recommendation Assessment Development and Education (GRADE) criteria [30–33, 43, 85] to assess the level of evidence for each CMD substrate and: (A) functional outcome, (B) neuro-physiologic measure, (C) tissue outcome. Final GRADE level was determined via consensus amongst the group of authors.

The GRADE level of evidence is split into four levels: A, B, C, and D. GRADE level A represents high evidence with multiple high-quality studies having consistent results. GRADE level B represents moderate evidence with one high-quality study, or multiple low-quality studies. GRADE level C evidence represents low evidence with one or more studies with severe limitations. Finally, GRADE level D represents very low evidence based on either expert opinion or few studies with severe limitations.

### Statistical analysis

A meta-analysis was not performed in this study due to the heterogeneity of data and study design within the articles identified.

## Results

### Search strategy results

The PRISMA flow diagrams showing the search strategy is available in Appendix A of the Supplementary materials. Overall, 2739 articles were identified, with 2716 from the database search and 23 from published meeting proceedings. There were 1590 duplicates removed, leaving 1149 references to review. Application of inclusion/exclusion criteria to the title and abstract of these articles resulted in selection of 159 articles for full manuscript review across all three review topics (Functional Outcome, (B) Neuro-physiologic Measures, and Tissue Outcome). One additional reference was added from the reference sections of review papers. A second filter of these 160 references provided 55 that met the final inclusion criteria for the Functional Outcome systematic review, 59 articles for inclusion the Neuro-physiologic Measures systematic review, and four articles for the Tissue Outcome systematic review.

We made substantial efforts to exclude duplicate patient data across studies. However, given that many of the papers came from centers of excellence for TBI research, some of the patient data may be cross reported in multiple studies. This would reduce the total overall number of unique patients. It was impossible, based on the information provided, to tease out all patients reported more than once.

### Patient/study demographics

#### Functional outcome studies

Of the 55 articles included in the review [2, 3, 6, 8–10, 12–14, 16–18, 20, 21, 25, 27–29, 34, 38, 42, 44, 45, 47, 48, 51, 53–55, 60–62, 64–66, 68–71, 73–76, 80, 81, 86, 88, 91–93, 99, 103, 105, 107, 108], 33 were formal manuscript publications and 22 were meeting abstract publications. There were 26 prospective studies, with three randomized control trials (RCT), 22 observational studies, and one single-arm study. Thirteen studies were retrospective series. Finally, 16 studies failed to clarify their study design [2, 3, 9, 14, 27, 28, 42, 45, 47, 60, 68, 76, 86, 103, 107, 108].

All but three studies [29, 42, 103] described populations on non-specified severe TBI patients, with no clear stratification based on primary brain injury pattern on admission neuroimaging. Two studies focused only on patients post-decompressive craniectomy (DC) [29, 103], with no clarification as to the severity/pattern of primary injury. Finally, one study focused only on patients with acute subdural hematomas (aSDH) [42]. Furthermore, details of the intensive care unit (ICU) treatments of these patients were not documented in most studies. Many studies mentioned institutional ICU protocols and ICP/CPP protocols, without giving details of therapies and individual variations in treatment. Furthermore, the application of barbiturate coma, therapeutic hypothermia, and DC were mentioned as potential therapies utilized, leading to significant variation in treatments received by individual patients.

Only three RCTs [2, 20, 65] and one prospective single arm study [44] provided details of the planned intervention. Post-TBI ICU care was mainly left unclear, stating “ICP/CPP directed protocols”, leaving room for significant procedural variation between individual patients.

Across all studies there were 2786 patients with severe TBI and assessment between common CMD measures and patient functional outcome. Forty-three patients were post-DC and 19 had aSDH. The remaining 2724 patients were described as severe-TBI patients without a detailed breakdown of injury patterns alongside CMD measures/functional outcomes. Table 1 displays a summary of the patient and study demographics.

**Table 1:** Study characteristics and patient demographics

Reference	Number of patients	Study type	Article location	Mean age (years)	Patient characteristics	Primary and secondary goal of study
Positive association studies						
Alessandri et al. [2]	54	Unknown	Meeting abstract	Unknown	Severe TBI	*Primary:* Determine the relationship between glutamate and lactate; determine link to patient outcome *Secondary:* None stated
Badenes et al. [6]	30	Prospective RCT	Meeting abstract	Unknown	Severe TBI; RCT comparing intensive glycemic control	*Primary:* Evaluate CMD-based changes with intensive glycemic control *Secondary:* Association of CMD markers with patient outcome
Belli et al. [8]	19	Prospective observational	Manuscript	41.5 years (range, 16–69)	Severe TBI	*Primary:* To determine the relationship between NAA, lactate, pyruvate, glycerol, and glutamate to outcome *Secondary:* Correlations between NAA and ICP/CPP/PbtO_2_; Correlations between various CMD markers
Bidot et al. [9]	12	Unknown	Meeting abstract	38 years (range, 18–70)	Severe TBI	*Primary:* Determine the link between CMD measures and neurological outcome *Secondary:* None mentioned
Bolcha et al. [10]	20	Prospective observational	Meeting abstract	Unknown	Severe TBI	*Primary:* Determine association between PbtO_2_ and CMD to patient outcome *Secondary:* None mentioned
Bullock et al. [12]	80	Prospective observational	Manuscript	Unknown	Severe TBI	*Primary:* Determine association of EAA and clinical outcomes (i.e., ischemic events, ICP/CPP, patient outcome) *Secondary:* None mentioned
Chamoun et al. [13]	135	Prospective observational	Manuscript	36.6 years (range, 16–91)	Severe TBI	*Primary:* Determine the association between CMD glutamate and prognosis *Secondary:* Correlation between glutamate and MABP/ICP/PbtO_2_/SjvO_2_
Chan et al. [14]	36	Unknown	Meeting abstract	Unknown	Severe TBI	*Primary:* To determine if the Pearson correlation between Glutamate/CPP correlated to outcome *Secondary:* None mentioned
Clausen et al. [17]	76	Prospective observational	Manuscript	Unknown	Severe TBI	*Primary:* Determine association between CMD glycerol and patient outcome *Secondary:* Determine association between CMD glycerol and monitoring (CPP/PbtO_2_)
Clausen et al. [18]	151	Prospective observational	Manuscript	35.5 years (range, unknown)	Severe TBI	*Primary:* Determine the association of brain pH, PCO_2_, lactate to patient outcome *Secondary:* Determine the association of above to CPP, PbtO_2_
Dizdarevic et al. [20]	30	Prospective RCT	Manuscript	45.8 years (range, 16–68)	Severe TBI	*Primary:* Determine impact of modified Lund therapy vs. standard CPP therapy in severe TBI *Secondary:* Determine the difference in CMD characteristics between the two therapies with regard to patient outcome
Figaji et al. [21]	5	Prospective observational	Meeting abstract	Unknown (children)	Severe TBI	*Primary:* Determine association of inflammatory cytokines and other CMD markers/patient outcome *Secondary:* None mentioned
Goodman et al. [25]	126	Prospective observational	Manuscript	34.4 years (range, unknown)	Severe TBI	*Primary:* Determine association between CMD lactate and glucose to ICP/CPP/PbtO_2_/SjvO_2_/rCBF *Secondary:* Clinical outcome
Gopinath et al. [27]	86	Unknown	Meeting abstract	Unknown	Severe TBI	*Primary:* Determine association between glutamate/aspartate and patient outcome *Secondary:* None mentioned
Gupta et al. [29]	25	Prospective observational	Manuscript	31.8 (range, 18–64)	Severe TBI with DC	*Primary:* Determine association between CMD/serum glucose measures pre- and post-DC and outcome
Gupta et al. [28]	41	Unknown	Meeting abstract	Unknown (over 18)	Severe TBI	*Primary:* Determine association between CMD glycerol and LPR with CPP and patient outcome *Secondary:* None mentioned
Hejcl et al. [34]	20	Prospective observational	Meeting abstract	Unknown	Severe TBI	*Primary:* Determine association between CMD/ICP/CPP/PbtO_2_ and patient outcome *Secondary:* None mentioned
Hutchinson et al. [38]	21	Prospective observational	Meeting abstract	Unknown	Severe TBI	*Primary:* Determine association of CMD measures and patient outcome *Secondary:* Not mentioned
Igarashi et al. [42]	19	Unknown	Meeting abstract	61.3 years (range, unknown)	Severe TBI with aSDH	*Primary:* Determine association between CMD measures and patient outcome
Johnston et al. [44]	11	Prospective single arm	Manuscript	32 years (range, unknown)	Severe TBI	*Primary:* To evaluate the relationship between ^15^O PET, PbtO_2_ and CMD to CPP augmentation in TBI *Secondary:* Determine association to outcome
Karathanou et al. [45]	33	Unknown	Meeting abstract	Unknown	Severe TBI	*Primary:* Determine association between CMD measures and patient outcome *Secondary:* None mentioned
Koura et al. [47]	83	Unknown	Meeting abstract	Unknown	Severe TBI	*Primary:* Determine association between CMD measures and CPP/ICP/patient outcome *Secondary:* None mentioned
Kurtz et al. [48]	46	Retrospective case series	Manuscript	55 years (range, unknown)	Severe TBI	*Primary:* Determine association between CMD/serum glucose and patient outcome *Secondary:* Association with CMD markers of metabolic stress
Li et al. [51]	53	Retrospective case series	Manuscript	42 years (range, 15–60)	Severe TBI	*Primary:* Determine association between CMD glycerol and ICP/CPP and patient outcome
Marcoux et al. [53]	15	Retrospective case series	Manuscript	30.1 years (range, 18–58)	Severe TBI	*Primary:* Determine association between CMD-based metabolic dysfunction and long-term tissue atrophy *Secondary:* Determine association with long-term outcome
Mazzeo et al. [54]	172	Retrospective case series	Manuscript	35.6 years (range, unknown)	Severe TBI	*Primary:* To validate a proposed ischemia score on outcome prediction in a retrospective cohort *Secondary:* Compare the ischemia score and CMD/PbtO_2_/CBF measurements
Mellergard et al. [55]	69	Retrospective case series	Manuscript	45.9 years (range, unknown)	Severe TBI	*Primary:* Determine potential age-related difference in CMD-based metabolic and inflammatory markers *Secondary:* CMD marker association to patient outcome
Nordstrom et al. [62]	213	Retrospective case series	Manuscript	40 years (range, 19–63)	Severe TBI	*Primary:* Determine correlation between CMD markers and injury patterns/patient outcome *Secondary:* None mentioned
Oddo et al. [64]	20	Retrospective case series	Manuscript	59 years (range, 36–76)	Severe TBI	*Primary:* To determine the impact of tight glycemic control on CMD markers *Secondary:* To determine the impact on patient outcome
Olivecrona et al. [65]	48	Prospective RCT	Manuscript	35.5 years (range, unknown)	Severe TBI – RCT comparing epoprostenol to placebo	*Primary:* 1. Determine the impact of epoprostenol on LPR, 2. Compare CMD between epoprostenol vs. placebo, 3. Correlate LPR to GOS at 3 months *Secondary:* None mentioned
Omerhodzic et al. [66]	33	Prospective observational	Manuscript	Unknown	Severe TBI	*Primary:* Determine the correlation between CMD markers and patient outcome *Secondary:* None mentioned
Paraforou et al. [69]	34	Prospective observational	Manuscript	43.6 years (range, unknown)	Severe TBI	*Primary:* Determine relationship between CPP/ICP/CMD and patient outcome *Secondary:* None mentioned
Peerdeman et al. [70]	15	Prospective observational	Manuscript	39.5 years (range, 18–69)	Severe TBI	*Primary:* Determine the association between secondary events (elevated ICP, low CPP, elevated temp) to CMD and outcome *Secondary:* None mentioned
Petzold et al. [71]	10	Prospective observational	Manuscript	39 years (range, 20–68)	Severe TBI	*Primary:* To correlate CMD measured neurofilament protein to injury pattern, physiologic measures, and outcome *Secondary:* Assess other CMD measures’ relationships to outcome
Reinert et al. [73]	20	Prospective observational	Manuscript	40.1 years (range, 21–70)	Severe TBI	*Primary:* Determine the correlation between autoregulation (PRx) and CPP/PbtO_2_/CMD and patient outcome *Secondary:* None mentioned
Reinert et al. [74]	85	Prospective observational	Manuscript	Unknown	Severe TBI	*Primary:* Determine the relationship between CMD potassium and ICP/CBF/other CMD substrates and patient outcome *Secondary:* None mentioned
Richards et al. [75]	9	Prospective observational	Manuscript	8 years (range, 3–14)	Severe TBI	*Primary:* Determine the association between glutamate and clinical outcome; also determine if an “excitotoxic index” (glutamate:glutamine) correlated to outcome *Secondary:* None mentioned
Robertson et al. [76]	79	Unknown	Meeting abstract	Unknown	Severe TBI	*Primary:* Determine the association between CMD glutamine/aspartate and patient outcome and PbtO_2_/CBF (TCD) *Secondary:* None mentioned
Sanchez-Porras et al. [81]	29	Retrospective case series	Manuscript	37.2 years (range, unknown)	Severe TBI	*Primary:* To evaluate long PRx and its relationship to patient outcome *Secondary:* To correlate PRx to other monitoring (i.e., CMD)
Sanchez et al. [80]	12	Unknown	Meeting abstract	Unknown	Severe TBI	*Primary:* Determine correlation between CDM measures and patient outcome *Secondary:* None mentioned
Singla et al. [86]	41	Unknown	Meeting abstract	Unknown	Severe TBI	*Primary:* Determine relationship between CPP/CMD to patient outcome *Secondary:* None mentioned
Stein et al. [88]	89	Prospective observational	Manuscript	46.4 years (range, 26–62)	Severe TBI	*Primary:* Determine the relationship between “metabolic crisis” (low glucose and elevated LPR) with outcome *Secondary:* None mentioned
Stiefel et al. [91]	27	Retrospective case series	Meeting abstract	Unknown	Severe TBI	*Primary:* Determine relationship between ICP/CPP/PbtO_2_/CMD and outcome *Secondary:* Determine if “metabolic compromise” (LPR > 25) or “metabolic crisis” (LPR > 40) is associated to outcome
Timofeev et al. [93]	223	Retrospective case series	Manuscript	Unknown	Severe TBI	*Primary:* Determine the relationship between CMD markers and neurological outcome *Secondary:* Association between CMD markers and ICP/Mx
Vespa et al. [99]	30	Prospective observational	Manuscript	34.4 years (range, unknown)	Severe TBI	*Primary:* To describe the trend of CMD glucose after TBI *Secondary:* Evaluate the lactate response to low CMD glucose; also assess FDG-PET response
Wang et al. [103]	18	Unknown	Meeting abstract	Unknown	Severe TBI – all post-DC	*Primary:* To evaluate CMD measures during medical ICP therapy, post-DC *Secondary:* None mentioned
Yokobori et al. [105]	25	Prospective observational	Manuscript	58.2 years (range, unknown)	Severe TBI	*Primary:* To determine the association between CMD markers and PRx within the first 7 days of injury *Secondary:* Determine association between CMD markers and patient outcome
Zauner et al. [107]	20	Unknown	Meeting abstract	Unknown	Severe TBI	*Primary:* To compare CMD markers, PbtO_2_, and CPP to patient outcome *Secondary:* None mentioned
Zauner et al. [108]	24	Prospective observational	Manuscript	Unknown	Severe TBI	*Primary:* To evaluate the association between PbtO_2_ and outcome *Secondary:* To evaluate the association between CMD markers and outcome
Nil association studies						
Alessandri et al. [3]	34	Unknown	Meeting abstract	Unknown	Severe TBI	*Primary:* To determine the association between CMD sodium and glutamate to outcome and ICP *Secondary:* None mentioned
Chen et al. [16]	20	Retrospective case series	Meeting abstract	Unknown (range, 18–90)	Severe TBI	*Primary:* To determine the association between CMD LPR/glucose with ICP/CPP in young and old patients *Secondary:* To determine the association between CMD markers and outcome
Nelson et al. [60]	26	Retrospective case series	Manuscript	52.2 years (range, 15–77)	Severe TBI	*Primary:* To determine patterns between CMD measures and ICP/CPP *Secondary:* Determine association between CMD markers and outcome
Nelson et al. [61]	90	Retrospective case series	Manuscript	48.9 years (range, 17–77)	Severe TBI	*Primary:* To determine the relationship between CMD measures and ICP/CPP *Secondary:* Determine association to outcome
Papanikolaou et al. [68]	30	Unknown	Meeting abstract	Unknown	Severe TBI	*Primary:* To determine the correlation between glycerol and other CMD measures to outcome and ICP/CPP *Secondary:* None mentioned
Thelin et al. [92]	14	Prospective observational	Manuscript	43.2 years (range, 20–62)	Severe TBI	*Primary:* To assess the relationship between CSF CMD and conventional brain parenchymal CMD *Secondary:* Determine association with outcome

*TBI* traumatic brain injury, *CMD* cerebral microdialysis, *RCT* randomized control trial, *ICP* intracranial pressure, *CPP* cerebral perfusion pressure, *NAA* *N*-acetyl acetate, *CSF* cerebrospinal fluid, *LPR* lactate:pyruvate ratio, *CBF* cerebral blood flow, *rCBF* regional cerebral blood flow, *SjvO*_*2*_ jugular venous oxygen saturation, *MABP* mean arterial blood pressure, *PbtO*_*2*_ partial pressure of oxygen in brain tissue, *Mx* autoregulation, *PRx* pressure reactivity monitoring, *TCD* transcranial Doppler, *PET* positron emission tomography, *FDG* fluorodeoxyglucose, *DC* decompressive craniectomy

#### Neuro-physiologic measures studies

Of the 59 articles included in this systematic review [1, 3–5, 7, 10–13, 15, 17, 18, 21, 25, 26, 28, 34, 39, 40, 44, 47, 48, 51, 52, 56–58, 60–63, 68–72, 74–76, 78, 79, 81–84, 86, 87, 89, 91, 93–101, 105, 109], 30 were prospective observational studies, four were prospective single-arm studies, one was a non-randomized controlled study, and 12 were retrospective case series. Twelve articles failed to provide enough information in order to determine the exact type of study conducted [3, 15, 28, 47, 68, 76, 79, 82, 84, 86, 89, 109] and were classified as “unknown”.

Many studies in this category focused on patients with severe TBI, without significant detail provided regarding the pattern of injuries suffered by individual patients (focal versus diffuse, etc). Furthermore, many studies failed to report CMD measures based on subcategories of severe TBI, only reporting mean values for the entire population studied. Two studies focused only on severe TBI patients whom died, and analyzed CMD measures during the herniation process [87, 96], and one study only included severe TBI patients on barbiturate therapy [26]. Descriptions of potential secondary insults and therapies were very limited in the majority of studies. Where such data were provided, details varied between studies, as did the therapies employed in each study.

Reference to ICP/CPP directed care/therapies was made in many studies, but the description as to how this was achieved was limited or absent. Many studies included significant variations in intensive care unit (ICU)-based therapies, including barbiturates [12, 18, 26] and therapeutic hypothermia [61]. The Lund concept for post-TBI ICU care was applied in some patients [62]. These limitations in data unavailability are unsurprising, since the aims and focus of the different studies was heterogeneous, and in general data were provided to address the specific (often pathophysiological) relationship that was being explored. However, these variations in disease course and physiology, coupled with variations in therapy and incomplete descriptions of the details of therapy made it impossible to undertake comprehensive exploration of the relationships between CMD variables and physiology or therapy.

A total of 2610 patients were studied across all included articles. The mean age varied across all studies, ranging from 8 years to 58.2 years. Twenty-three studies failed to disclose the age of the patients [3, 10, 12, 15, 17, 21, 28, 34, 47, 58, 68, 72, 74, 76, 83, 84, 86, 89, 91, 93, 94, 96, 109]. Only four studies reported the inclusion of a small number of pediatric patients (age under 16) [51, 56, 61], with only one study clearly displaying the number of pediatric patients [75]. Overall, the total number of pediatric patients is quite small. Some studies included moderate TBI patients with the severe cohorts. It was impossible to separate the two groups. As such, the compiled patient data, both moderate and severe TBI, were included within this review. Study and patient characteristics can be seen in Table 2.

**Table 2:** Neurophysiologic measure studies: study characteristics and patient demographics

Reference	Number of patients	Study type	Article location	Mean age (years)	Patient characteristics	Primary and secondary goal of study
ICP/CPP positive association studies						
Adamides et al. [1]	14	Prospective observational	Manuscript	40 years (range, 19–66)	Severe TBI	*Primary:* To evaluate the temporal relationship between CMD measures and ICP/CPP *Secondary:* None mentioned
Belli et al. [7]	25	Prospective observational	Manuscript	41.8 years (range, 16–78)	Severe TBI	*Primary:* To evaluate the relationship between CMD measures and ICP *Secondary:* None mentioned
Bolcha et al. [10]	20	Prospective observational	Meeting abstract	Unknown	Severe TBI	*Primary:* Determine association between ICP/PbtO_2_ and CMD to patient outcome *Secondary:* None mentioned
Bullock et al. [12]	80	Prospective observational	Manuscript	Unknown	Severe TBI	*Primary:* Determine association of EAA and clinical outcomes (i.e., ischemic events, ICP/CPP, patient outcome) *Secondary:* None mentioned
Clausen et al. [17]	76	Prospective observational	Manuscript	Unknown	Severe TBI	*Primary:* Determine association between CMD glycerol and patient outcome *Secondary:* Determine association between CMD glycerol and monitoring (CPP/PbtO_2_)
Clausen et al. [18]	151	Prospective observational	Manuscript	35.5 years (range, unknown)	Severe TBI	*Primary:* Determine the association of brain pH, PCO_2_, lactate to patient outcome *Secondary:* Determine the association of above to CPP, PbtO_2_
Goodman et al. [26]	7	Retrospective case series	Manuscript	34.6 years (range, 19–48)	Severe TBI on barbiturate therapy	*Primary:* To evaluate the impact of barbiturate sedation on CMD measures and ICP in severe TBI *Secondary:* None mentioned
Goodman et al. [25]	126	Prospective observational	Manuscript	34.4 years (range, unknown)	Severe TBI	*Primary:* Determine association between CMD lactate and glucose to ICP/CPP/PbtO_2_/SjvO_2_/rCBF *Secondary:* Clinical outcome
Gupta et al. [28]	41	Unknown	Meeting abstract	Unknown (over 18)	Severe TBI	*Primary:* Determine association between CMD glycerol and LPR with CPP and patient outcome *Secondary:* None mentioned
Hejcl et al. [34]	20	Prospective observational	Meeting abstract	Unknown	Severe TBI	*Primary:* Determine association between CMD/ICP/CPP/PbtO_2_ and patient outcome *Secondary:* None mentioned
Koura et al. [47]	83	Unknown	Meeting abstract	Unknown	Severe TBI	*Primary:* Determine association between CMD measures and CPP/ICP/patient outcome *Secondary:* None mentioned
Kurtz et al. [48]	46	Retrospective case series	Manuscript	55 years (range, unknown)	Severe TBI	*Primary:* Determine association between CMD/serum glucose and patient outcome *Secondary:* Association with CMD markers of metabolic stress (CPP and PbtO_2_)
Li et al. [51]	53	Retrospective case series	Manuscript	42 years (range, 15–60)	Severe TBI	*Primary:* Determine association between CMD glycerol and ICP/CPP and patient outcome *Secondary:* None mentioned
Meixensberger et al. [56]	9	Prospective observational	Manuscript	33.3 years (range, 14–60)	Severe TBI	*Primary:* To determine the correlation between CMD measures and ICP/CPP and PbtO_2_ *Secondary:* None mentioned
Nelson et al. [61]	90	Retrospective case series	Manuscript	48.9 years (range, 17–77)	Severe TBI	*Primary:* To determine the relationship between CMD measures and ICP/CPP *Secondary:* Determine association to outcome
Nordstrom et al. [62]	213	Retrospective case series	Manuscript	40 years (range, 19–63)	Severe TBI	*Primary:* Determine correlation between CMD markers and injury patterns/patient outcome *Secondary:* None mentioned
Papanikolaou et al. [68]	30	Unknown	Meeting abstract	Unknown	Severe TBI	*Primary:* To determine the correlation between glycerol and other CMD measures to outcome and ICP/CPP *Secondary:* None mentioned
Paraforou et al. [69]	34	Prospective observational	Manuscript	43.6 years (range, unknown)	Severe TBI	*Primary:* Determine relationship between CPP/ICP/CMD and patient outcome *Secondary:* None mentioned
Richards et al. [75]	9	Prospective observational	Manuscript	8 years (range, 3–14)	Severe TBI	*Primary:* Determine the association between glutamate and clinical outcome; also determine if an “excitotoxic index” (glutamate:glutamine) correlated to outcome *Secondary:* Correlation of CMD markers to ICP
Salci et al. [79]	10	Unclear	Manuscript	45.1 years (range, 19–63)	Severe TBI	*Primary:* To determine the relationship between CMD measures and ICP/compliance *Secondary:* None mentioned
Sarrafzadeh et al. [82]	18	Unclear	Meeting abstract	36 years (range, unknown)	Severe TBI	*Primary:* To determine the relationship between CMD measures and ICP/SjvO_2_/PbtO_2_ *Secondary:* None mentioned
Singla et al. [86]	41	Unknown	Meeting abstract	Unknown	Severe TBI	*Primary:* Determine relationship between CPP/CMD to patient outcome *Secondary:* None mentioned
Stahl et al. [87]	7	Retrospective case series	Manuscript	55.4 years (range, 27–74)	Severe TBI whom died	*Primary:* To document CMD measure responses to ICP during end of life *Secondary:* None mentioned
Stein et al. [89]	117	Unclear	Meeting abstract	Unknown	Severe TBI	*Primary:* To determine the relationship between CMD measured metabolic crisis (LPR > 40) and ICP *Secondary:* None mentioned
Stiefel et al. [91]	27	Retrospective case series	Meeting abstract	Unknown	Severe TBI	*Primary:* Determine relationship between ICP/CPP/PbtO_2_/CMD and outcome *Secondary:* Determine is “metabolic compromise” (LPR > 25) or “metabolic crisis” (LPR > 40) is associated to outcome
Timofeev et al. [93]	223	Retrospective case series	Manuscript	Unknown	Severe TBI	*Primary:* Determine the relationship between CMD markers and neurological outcome *Secondary:* Association between CMD markers and ICP/PRx
Timofeev et al. [94]	97	Prospective observational	Manuscript	Unknown	Severe TBI	*Primary:* To determine the relationship between CMD measures and PRx, ICP/CPP, PbtO_2_ *Secondary:* None mentioned
Vespa et al. [98]	17	Prospective observational	Manuscript	39.4 years (range, unknown)	Severe TBI	*Primary:* To determine the extent and duration of CMD glutamate changes post-TBI *Secondary:* To determine the correlation between glutamate and CPP
PbtO_2_/SjvO_2_ positive association studies						
Chan et al. [15]	25	Unknown	Meeting abstract	Unknown	Severe TBI	*Primary:* To determine SjvO_2_ correlated to CMD measures in TBI *Secondary:* None mentioned
Figaji et al. [21]	5	Prospective observational	Meeting abstract	Unknown (children)	Severe TBI	*Primary:* Determine association of inflammatory cytokines and other CMD markers/patient outcome *Secondary:* None mentioned
Menzel et al. [57]	24	Prospective Non-randomized	Manuscript	34.4 years (range, 20–74)	Severe TBI Non-randomized to high FiO_2_	*Primary:* To determine the impact of increased FiO_2_ on CMD measures and PbtO_2_ *Secondary:* None mentioned
Menzel et al. [58]	14	Prospective single arm	Manuscript	Unknown (adults)	Severe TBI	*Primary:* To determine the change in CMD markers, CBF, and PbtO_2_ to induced hyperoxia *Secondary:* None mentioned
Nortje et al. [63]	11	Prospective single arm	Manuscript	42 years (range, 17–64)	Severe TBI	*Primary:* To determine if normobaric hyperoxia leads to changes in ^15^O PET, CMD and PbtO_2_ *Secondary:* None mentioned
Purins et al. [72]	23	Prospective observational	Manuscript	Unknown	Severe TBI	*Primary:* To determine the relationship between PbtO_2_ and CMD measures *Secondary:* None mentioned
Robertson et al. [76]	79	Unknown	Meeting abstract	Unknown	Severe TBI	*Primary:* Determine the association between CMD glutamine/aspartate and patient outcome and PbtO_2_/CBF (TCD) *Secondary:* None mentioned
Sarrafzadeh et al. [83]	35	Retrospective case series	Manuscript	Unknown	Severe TBI	*Primary:* To determine the relationship between CMD and PbtO_2_ *Secondary:* None mentioned
Sarrafzadeh et al. [84]	24	Unknown	Meeting abstract	Unknown	Severe TBI	*Primary:* To determine the relationship between CMD and PbtO_2_ *Secondary:* None mentioned
Timofeev et al. [95]	56	Prospective observational	Manuscript	38.5 years (range, 25–52)	Severe TBI	*Primary:* To compare brain pH to disturbances in PbtO_2_ and CMD *Secondary:* None mentioned
Valdaka et al. [96]	5	Prospective observational	Manuscript	Unknown	Severe TBI during herniation and death	*Primary:* To evaluate the correlation between CMD and PbtO_2_ in fatal TBI *Secondary:* None mentioned
Vilalta et al. [101]	30	Prospective single arm	Manuscript	27 years (range, 17–59)	Severe TBI - Normobaric hyperoxic challenge	*Primary:* To determine the impact of normbaric hyperoxia on CMD and PbtO_2_ in TBI *Secondary:* Determine the impact on outcome
Autoregulation - positive association studies						
Asgari et al. [4]	19	Prospective observational	Manuscript	41.9 years (range, unknown)	Severe TBI	*Primary:* To evaluate the relationship between MOCAIP ICP waveform-based autoregulation measurements and CMD measures *Secondary:* None mentioned
Asgari et al. [5]	30	Prospective observational	Manuscript	46 years (unknown)	Severe TBI	*Primary:* To determine the relationship between CMD measures and PMTM ICP waveform analysis of vasoconstriction/vasodilation *Secondary:* None mentioned
Yokobori et al. [105]	25	Prospective observational	Manuscript	58.2 years (range, unknown)	Severe TBI	*Primary:* To determine the association between CMD markers and PRx within the first 7 days of injury *Secondary:* Determine association between CMD markers and patient outcome
Imaging-based positive association studies						
Bouzat et al. [11]	27	Prospective observational	Manuscript	39 years (range, 24–54)	Severe TBI	*Primary:* To determine the relationship between CMD, PbtO_2_ and ICP measures to CBF as measured via CTP *Secondary:* None mentioned
Hutchinson et al. [39]	13	Prospective observational	Manuscript	34 years (range, 16–66)	Severe TBI	*Primary:* To determine relationship between CMD measures and H_2_^15^O PET *Secondary:* None mentioned
Hutchinson et al. [40]	17	Prospective observational	Manuscript	43.4 years (range, 17–66)	Severe TBI	*Primary:* To determine the relationship between CMD measures and FDG PET *Secondary:* None mentioned
Reinert et al. [74]	85	Prospective observational	Manuscript	Unknown	Severe TBI	*Primary:* Determine the relationship between CMD potassium and ICP/CBF/other CMD substrates and patient outcome *Secondary:* None mentioned
Sala et al. [78]	24	Prospective observational	Manuscript	36 years (range, unknown)	Severe TBI	*Primary:* To determine the relationship between CMD measures and CBF post-TBI *Secondary:* None mentioned
Vespa et al. [97]	19	Prospective observational	Manuscript	32.3 years (range, 16–57)	Severe TBI	*Primary:* To evaluation the presence of CMD measures during changes in CMRO_2_ and CMRglu *Secondary:* None mentioned
Vespa et al. [99]	30	Prospective observational	Manuscript	34.4 years (range, unknown)	Severe TBI	*Primary:* To describe the trend of CMD glucose after TBI *Secondary:* Evaluate the lactate response to low CMD glucose; also assess FDG-PET and Xe CT CBF response
Zauner et al. [109]	25	Unknown	Meeting abstract	Unknown	Severe TBI	*Primary:* To evaluate the relationship between CMD glutamate and CBF as measured by Xe CT *Secondary:* None mentioned
Negative association studies						
Alessandri et al. [3]	34	Unknown	Meeting abstract	Unknown	Severe TBI	*Primary:* To determine the association between CMD sodium and glutamate to outcome and ICP *Secondary:* None mentioned
Chamoun et al. [13]	135	Prospective observational	Manuscript	36.6 years (range, 16–91)	Severe TBI	*Primary:* Determine the association between CMD glutamate and prognosis *Secondary:* Correlation between glutamate and MABP/ICP/PbtO_2_/SjvO_2_
Johnston et al. [44]	11	Prospective single arm	Manuscript	32 years (range, unknown	Severe TBI	*Primary:* To evaluate the relationship between ^15^O PET, PbtO_2_ and CMD to CPP augmentation in TBI *Secondary:* Determine association to outcome
Nelson et al. [60]	26	Retrospective case series	Manuscript	52.2 years (range, 15–77)	Severe TBI	*Primary:* To determine patterns between CMD measures and ICP/CPP *Secondary:* Determine association between CMD markers and outcome
Peerdeman et al. [70]	15	Prospective observational	Manuscript	39.5 years (range, 18–69)	Severe TBI	*Primary:* Determine the association between secondary events (elevated ICP, low CPP, elevated temp) to CMD and outcome *Secondary:* None mentioned
Petzold et al. [71]	10	Prospective observational	Manuscript	39 years (range, 20–68)	Severe TBI	*Primary:* To correlate CMD measured neurofilament protein to injury pattern, physiologic measures, and outcome *Secondary:* Assess other CMD measures relation to outcome
Sanchez-Porras et al. [81]	29	Retrospective case series	Manuscript	37.2 years (range, unknown)	Severe TBI	*Primary:* To evaluate long PRx and its relationship to patient outcome *Secondary:* To correlated PRx to other monitoring (i.e., CMD)
Vespa et al. [100]	21	Retrospective case series	Manuscript	31.4 years (range, 18–58)	Severe TBI	*Primary:* To determine the relationship between CPP and LPR *Secondary:* None mentioned

*TBI* traumatic brain injury, *GOS* Glasgow outcome scale, *GOSE* Glasgow outcome scale extended, *CMD* cerebral microdialysis, *RCT* randomized control trial, *ICP* intracranial pressure, *CPP* cerebral perfusion pressure, *NAA* *N*-acetyl acetate, *CSF* cerebrospinal fluid, *LPR* lactate:pyruvate ratio, *CBF* cerebral blood flow, *rCBF* regional cerebral blood flow, *SjvO*_*2*_ jugular venous oxygen saturation, *MABP* mean arterial blood pressure, *PbtO*_*2*_ partial pressure of oxygen in brain tissue, *Mx* autoregulation, *PRx* pressure reactivity monitoring, *TCD* transcranial Doppler, *PET* positron emission tomography, *FDG* fluorodeoxyglucose, *OEF* oxygen extraction fraction, *ROI* region of interest, *CMRglc* cerebral metabolic rate of glucose consumption, *CMRO*_*2*_ cerebral metabolic rate of oxygen consumption, *Xe CT* xenon-enhanced computed tomography, *CTP* compute tomographic perfusion imaging, *NIRS* near-infrared spectroscopy, *MMM* multi-modal monitoring, *DC* decompressive craniectomy

#### Tissue outcome studies

Three of the four articles included in the tissue outcome systematic review [22, 23, 53, 89] were prospective observational studies [22, 23, 89] and one was a retrospective case series [53]. All studies focused on the severe TBI patient population with no specific subcategory of interest declared.

A total of 52 patients were studied. The age of the patients studied was mentioned only in one study [53], where it was reported as a mean of 30.1 years (range, 18–58 years). No specific details of patient comorbidities, acute phase treatment of ICP/CPP, and complications related to critical illness were report in any of the studies included in the review. Study and patient demographics can be seen in Table 3.

**Table 3:** Study characteristics and patient demographics

Reference	Number of patients	Study type	Article location	Mean age (years)	Patient characteristics	Primary and secondary goal of study
Positive association studies						
Filippou et al. [23]	6	Prospective observational	Meeting abstract	Unknown	Severe TBI	*Primary:* CMD measures and MRS/ADC imaging *Secondary:* None stated
Marcoux et al. [53]	15	Retrospective case series	Manuscript	30.1 years (range, 18–58)	Severe TBI	*Primary:* Determine association between CMD-based metabolic dysfunction and long-term tissue atrophy *Secondary:* Determine association with long-term outcome
Nil association studies						
Filippou et al. [22]	10	Prospective observational	Meeting abstract	Unknown	Severe TBI	*Primary:* Determine CMD measure correlation to MRS/ADC *Secondary:* None mentioned
Stein et al. [89]	21	Prospective observational	Meeting abstract	Unknown	Severe TBI	*Primary:* To determine if CMD defined metabolic crisis (LPR > 25; glucose <0.8 mmol/l) correlates with MRI-based ADC changes

*TBI* traumatic brain injury, *CMD* cerebral microdialysis, *LPR* lactate:pyruvate ratio, *MRS* magnetic resonance spectroscopy, *MRI* magnetic resonance imaging, *ADC* apparent diffusion coefficient

### CMD measurement technique

Details of the use of CMD monitoring were present in many studies. The most commonly quoted sampling intervals ranged from 30 min to hourly. Unfortunately, the use of pooled sample analysis was not always clear. Consequently, despite hourly sampling, the data in most manuscripts did not allow us to exclude the possibility that samples may have been pooled over longer periods and analyzed for substrates over these integrated time intervals.

The most commonly reported CMD measures included: glucose, lactate, pyruvate, LPR, ± glutamate. Some studies reported CMD measure of only one substrate, typically glutamate or lactate.

A summary of the CMD measurement techniques for the functional outcome, neuro-physiologic measure, and tissue outcome studies can be seen in Appendix C of the Supplementary materials. Furthermore, a study-by-study tabulation of CMD measurement details can be seen in Appendices E, F, and G of the Supplementary materials.

### Measures of outcome

A summary of the various techniques employed in the measurement of functional outcome, neuro-physiologic measures and tissue outcome can be seen within Appendix D of the supplementary materials. Furthermore, a study-by-study tabulation of the outcome measure documented can be seen in Appendices E, F, and G of the Supplementary materials.

### Patient functional outcome - positive association studies

#### Glucose

Thirteen studies documented an association between CMD measured glucose and mortality/Glasgow Outcome Scale (GOS) at 3–6 months [6, 9, 29, 38, 48, 54, 64, 66, 73, 80, 105, 107, 108]. The documented association was that low CMD glucose was associated with patient functional outcome. The mean value for “low” CMD glucose varied across studies (range, 0.46–1.39 mmol/l), with these values being significantly lower than the “good” outcome cohorts. Of note, one study quoted a mean value of 13.8 mmol/l in the poor outcome group within a table, however we suspect this was a typo in units (i.e., mmol/l instead of mg/l).

One study reported an association between high lactate:glucose ratio (LGR) to outcome [54]. In addition, one study reported an association between low brain:serum glucose levels (<0.12) and poor patient outcome [48].

#### Glutamate

Fourteen studies documented an association between CMD-measured glutamate levels and patient outcome as assessed by mortality and GOS at 3–6 months [2, 12–14, 27, 38, 47, 55, 75, 76, 81, 86, 93, 105]. A threshold of glutamate greater than 20 µmol/l was reported in many of these studies to be associated with mortality and poor GOS at 3–6 months post-injury.

#### Glycerol

Fourteen studies reported an association between CMD measured glycerol and patient outcome, measured as mortality and/or GOS at 3–6 months [8, 10, 17, 20, 28, 34, 42, 45, 51, 54, 69, 70, 93, 105]. There was a documented statistically significant association between elevated mean CMD glycerol levels and increased mortality/poor outcome at 3–6 months [12–14, 27]. The reported ranges for “high” CMD glycerol varied substantially across studies, with the “poor” outcome cohorts displaying mean levels ranging from 83 µmol/l to over 150 µmol/l.

#### Lactate/LPR

Twenty-five studies reported an association between CMD-measured LPR and patient functional outcome, as assessed by mortality and GOS at 3–6 months [8–10, 20, 21, 28, 34, 38, 42, 44, 45, 53, 55, 62, 65, 69, 71, 80, 86, 88, 92, 93, 99, 103, 105]. All studies documented a significant association between elevated mean LPR (or baseline admission LPR) and increased mortality/poor GOS at 3–6 months. The threshold values reported for poor outcome varied significantly, ranging from 25 up to and exceeding 80. The most commonly quoted LPR thresholds for poor outcome ranged between 25 and 40.

Fourteen studies reported separate associations between the mean CMD lactate concentration and increased mortality and/or poor outcome at 3–6 months [2, 9, 18, 25, 38, 42, 54, 73, 80, 81, 93, 99, 107, 108]. Only one study made direct comments about pyruvate levels and associated mortality/morbidity. This study reported that sustained mean pyruvate levels below 70 µmol/l, with LPR > 30, are associated with mortality at 6 months [62].

#### Potassium

Only one study was found describing the association between CMD potassium and patient outcome, as assessed via a dichotomized GOS at 3 months [74]. This study documented an association between mean potassium levels less than 1.8 mmol/l and good outcome at 3 months (*p* < 0.0001). The measurement of potassium levels in recovered microdialysate can be challenging as the perfusion fluid often contains potassium.

### Neuro-physiologic measures – positive association studies

#### CMD association with ICP/CPP

Overall, there were 1684 patients described within studies which found positive association between one or more common CMD measures and ICP and/or CPP.

The mean LPR was found to be associated with ICP/CPP in 15 studies [1, 7, 10, 28, 34, 61, 62, 69, 79, 86, 87, 89, 93]. Seven studies displayed an association between elevated ICP and an increase in LPR [7, 10, 69, 79, 87, 89, 93]. Four studies displayed an association between low CPP and elevated LPR [28, 62, 86, 91]. Finally, four studies described an association between elevated ICP/low CPP and increased LPR [1, 34, 61, 94]. Various LPR thresholds were described for these relationships, with and LPR of greater than 25 to 40 being the most commonly quoted.

Elevated mean glutamate was found to be associated with ICP/CPP in 11 studies [1, 7, 12, 26, 47, 56, 75, 82, 87, 93, 98]. Elevated mean glutamate was seen during episodes of increased ICP in eight studies [7, 26, 47, 56, 75, 82, 87, 91], and increased ICP/low CPP in three studies [1, 12, 98]. Glutamate thresholds for this association were seldom reported, with only one study reporting levels greater than 20 µmol/l as statistically associated with ICP/CPP [12].

Elevated mean glycerol was found to be associated with ICP/CPP in 12 studies [1, 7, 10, 17, 34, 48, 61, 68, 69, 86–88]. Increased mean glycerol was seen during episodes of increased ICP in seven studies [7, 10, 34, 68, 69, 87, 89], low CPP in two studies [17, 86], and elevated ICP/low CPP in three studies [1, 51, 61]. Like glutamate, glycerol thresholds seen during these associations were not commonly described, with only one study reporting a threshold of 100 µmol/l [7].

Some studies described relationships between common CMD markers and ICP/CPP that were less robust. Mean CMD lactate elevations, in isolation, were found to be associated with elevated ICP and/or low CPP in only four studies [18, 25, 61, 78]. Low mean glucose was associated with elevated ICP and/or low CPP in three studies [25, 48, 56]. Finally, high potassium levels were associated with ICP elevations in one study [74].

#### CMD association with SjvO_2_

Three studies referred to some association between common CMD measures and SjvO_2_, totaling 169 patients [15, 25, 82]. Elevated mean lactate was found to be associated with low SjvO_2_ in three studies [15, 25, 82]. Increased mean glutamate and glycerol were also found to be associated with low SjvO_2_ in two studies [15, 82].

#### CMD association with PbtO_2_

Eighteen studies made some reference to a positive association between one or more common CMD measures and PbtO_2_, totaling 746 patients [10, 17, 18, 21, 34, 47, 56, 58, 63, 72, 76, 82–84, 94–96, 101]. Location of the PbtO_2_ probe is believed to have been near the CMD probe in all studies, though this was not explicitly stated in the majority. Low PbtO_2_ was found to be associated with elevated mean LPR in 11 studies [10, 15, 34, 48, 56, 63, 72, 94–96, 101], elevated mean glycerol in three studies [17, 72, 82], elevated mean glutamate in five studies [72, 76, 82, 83, 96], elevated mean lactate alone in seven studies [18, 21, 58, 82, 95, 96, 101], low mean glucose in three studies [48, 85, 101], and high mean potassium in one study [96].

#### CMD association to autoregulation

An association between autoregulation measures and common CMD measures was reported in five studies, totaling 394 patients [4, 5, 93, 94, 105]. Pressure reactivity index (PRx) monitoring was utilized in three studies, with an association between worsening autoregulation and elevated mean LPR [93, 94, 105], glutamate [93], glycerol [93], and glucose [105]. Two studies reported autoregulation measurement via ICP computational waveform analysis, with an association between poor autoregulation and elevated LPR [4, 5].

#### CMD associations with cerebral blood flow and metabolism

Ten studies reported an association between CMD analytes and measures of cerebral blood flow using a variety of techniques (PET, CTP, Xe CT, TCD (transcranial Doppler) or laser Doppler flowmeter), totaling 372 patients [11, 39, 40, 51, 74, 76, 78, 99, 109].

Two studies reported a correlation between LPR and ^15^O PET measurements, with the following relationships described: positive correlation between LPR and oxygen extraction fraction (OEF) [39], and a negative correlation between LPR and cerebral metabolic rate of oxygen consumption (CRMO_2_) [97]. The ROI for these PET studies was near the CMD catheter. A CTP-based assessment of CBF was described in five studies. Two studies described CTP based decreases in CBF in association with low glucose/elevated LPR [11] and glycolytic lactate elevations [78]. Three studies utilized Xe CT, detailing an association between elevated mean potassium [74], elevated mean glutamate [108], and decreased mean glucose [99] with reduced CBF. Transcranial Doppler ultrasonography was applied in two studies. One described TCD based decrease in CBF velocities associated with elevated mean glutamate. The second study detailed laser Doppler flowmeter assessment of CBF, with an association between elevated glycerol and low CBF identified.

Two studies reported an association between fluorodeoxyglucose (FDG) PET and common CMD measures, with the following relationships: increased lactate/pyruvate positively correlated to cerebral metabolic rate of glucose consumption (CRM_GLC_) [40], and low glucose correlated to increased FDG uptake [99].

### Tissue outcome – positive association studies

Two studies included within the review were identified as “positive” studies, in that an association between CMD measures and tissue fate were identified. Details can be seen in Table 3.

One of these studies was a meeting abstract describing the use of MRS and MR diffusion tensor imaging (DTI) at an undisclosed interval in the acute/subacute phase post-injury [23]. The MRS analysis focused on *N*-acetyl aspartate (NAA), choline (Cho), and creatinine (Cr) peaks, in addition to apparent diffusion coefficient (ADC) on MRI imaging. The goal was to identify possible areas of ischemia/infarction using ADC thresholds and identify areas of neuronal loss on MRS measures. The region of interest (ROI) for the MRS voxel target was around the area of the CMD probe, which was in an unspecified location (with radiologically normal (no structural injury on CT) versus lesional tissue). The location of the “control” voxel was unclear in these patients. The study demonstrated a negative correlation between CMD LPR and MRS defined NAA (*p* = 0.037) and Cr peaks (*p* < 0.001). In addition, CMD measured glucose was positively correlated with the Cho peak (*p* = 0.007) and NAA/Cho ratio (*p* = 0.028).

The second “positive” study focused on long-term assessment of chronic frontal lobe atrophy at 6 months post-injury [53]. This article utilized an MRI volumetric based assessment of frontal lobe atrophy and attempted to determine an association with acute/subacute CMD measures from “healthy tissue”. It was found that the degree of LPR elevation and the duration of abnormal LPR were positively associated with frontal lobe atrophy at 6 months (*p* < 0.01). In addition, it was noted that the percentage of time spend with LPR values greater than 40 directly correlated to frontal lobe atrophy at 6 months.

### Nil association studies

Overall, for the majority of the studies identified documented positive associations between one or more CMD analyte and the various outcomes of interest. A minority of studies, and thus the minority of the overall patient population documented within the literature, reported a “nil” association between a specific CMD analyte and outcome (i.e., functional, neuro-physiologic, or tissue). A summary of the “nil” association studies can be found within Appendix H of the Supplementary materials. Furthermore, a study-by-study breakdown of the “nil” association studies can be found in Appendices E, F, and G of the Supplementary materials.

### Complications of CMD monitoring

#### Functional outcome studies

Many studies failed to document complications/harms associated with CMD monitoring. Only four studies mentioned documentation of complications [8, 12, 65, 99]. Two studies stated “no complications” [65, 99]. One study documented a focal intra-cerebral hemorrhage (ICH) during insertion which required surgical evacuation [4]. Finally, one study described three instances of CMD catheter malfunction, requiring re-insertion of a new catheter [12].

#### Neuro-physiologic measure studies

Only five studies reported complications related to CMD monitoring [7, 12, 56, 98, 99]. Three studies reported “no complications” related to CMD [7, 98, 99]. Two studies reported CMD catheter malfunction in a total of five patients [12, 56].

#### Tissue outcome studies

All studies identified failed to disclose any complications related to CMD monitoring.

### Assessment of bias

The risk of bias was assessed via the RTI item bank [102], with the results tabulated in Appendix B of the supplementary material. Overall, all meeting abstracts included within these systematic reviews were deemed very high risk in almost all categories in the RTI bank. A short descriptive reasoning for the bias assessments can also be found in Appendix B.

### Level of evidence

#### Functional outcome

As previously outlined, the level of evidence was assessed as per GRADE criteria through consensus amongst the authors. Given the overall high bias risk and shortcomings of all the studies included within the systematic review, the level of evidence ranges from low to very low in support of an association between common CMD measures and patient outcome as assessed by mortality and GOS at 3–6 months. A summary can be seen in Table 4.

**Table 4:** GRADE level of evidence for CMD measures and patient functional outcome

CMD measure	Association with patient functional outcome	GRADE level of evidence
Glucose	Low mean glucose is associated with poor outcome/mortality at 3–6 months post-injury	C (Low) – Large number of studies with significant limitations
Glutamate	High mean glutamate is associated with poor outcome/mortality at 3–6 months post-injury	C (Low) – Large number of studies with significant limitations
Glycerol	High mean glycerol is associated with poor outcome/mortality at 3–6 months post-injury	C (Low) – Large number of studies with significant limitations
LPR	Elevated mean LPR (>25 to 40) is associated with poor outcome/mortality at 3–6 months post-injury	C (Low) – Large number of studies with significant limitations
Lactate	Elevated mean lactate is associated with poor outcome/mortality at 3–6 months post-injury	C (Low) – Large number of studies with significant limitations
Pyruvate	Persistently low mean pyruvate levels, in the presence of elevated LPR, is associated with üoor outcome	D (Very Low) – limited number of studies evaluating pyruvate in isolation; significant limitations identified within these studies
Potassium	Mean potassium levels less than 1.8 mmol/l is associated with a good GOS at 3 months post-injury	D (Very Low) – one study with limitations
Sodium	There is no association between mean CMD sodium levels and outcome	D (Very Low) – one study with limitations

*CMD* cerebral microdialysis, *LPR* lactate:pyruvate ratio, *mmol* millimolar, *l* liter, *GOS* Glasgow Outcome Scale

#### Neuro-physiologic measures

Overall level of evidence for the common CMD measures and their relationship to physiologic measures were assessed via the GRADE criteria. This assessment showed that most CMD biomarkers displayed very low evidence in support of a relationship with specific physiologic measures. Given the limitations listed above, we were limited in the strength of conclusions that we provide.

There currently exists GRADE C level of evidence supporting an association between CMD measured LPR, glutamate and glycerol with ICP and/or CPP. In addition, there currently exists GRADE C evidence to support an association between LPR and PbtO_2_. All remaining CMD measures displayed either GRADE D evidence, or occasionally no evidence, to support a relationship with physiologic measures. A tabulated summary of the GRADE evidence can be seen in Table 5.

**Table 5:** GRADE level of evidence for association between CMD analytes and physiologic measures

Physiologic measure	GRADE level of evidence	CMD measure(s)	Rationale
ICP/CPP	C (low)	LPR, glutamate, glycerol	Several studies with large patient numbers and statistically significant relations but major study limitations in all
	D (very low)	Lactate (alone), pyruvate (alone), glucose, potassium	Small number of studies with severe limitations
SjvO_2_	D (very low)	Lactate (alone), glutamate, glycerol	Very limited number of studies with significant bias/limitation
PbtO_2_	C (low)	LPR	Several studies with severe limitations
	D (very low)	Glycerol, glutamate, lactate, potassium glucose	Very limited number of studies. All significantly flawed. Publication bias high in this area.
Autoregulation (PRx and ICP waveform analysis)	D (very low)	LPR, glutamate, glycerol	Very limited number of studies. Bias high. Publication bias very high.
Imaging	No GRADE applied	N/A	This area of the literature was extremely scarce, heterogeneous, suffering from publication bias, design bias. Thus, we felt it inappropriate to assign any GRADE level to this area at this time

*ICP* intracranial pressure, *CPP* cerebral perfusion pressure, *CMD* cerebral microdialysis, *SjvO*_*2*_ jugular venous oxygen saturation monitoring, *PbtO*_*2*_ brain tissue oxygen level monitoring, *PRx* pressure reactivity index monitoring, *LPR* lactate:pyruvate ratio

#### Tissue outcome

Given the significant limitations in each study based on design and bias risk assessment, it was difficult to assign a formal GRADE level of evidence. Furthermore, there were studies both in support and against any correlation between CMD measures and tissue outcome. Thus, we are left with the following conclusions.

There exists GRADE D (very low) evidence to suggest an association between CMD measured LPR greater than 40 in the acute/subacute phase and frontal lobe atrophy at 6 months post-injury.

There is unclear evidence to suggest a correlation between CMD measures and MRS or ADC-based tissue outcome assessment, with both low-quality evidence to support and refute an association at this time.

## Discussion

We believe that this three-stage systematic review includes the major available studies documenting the relationship between common CMD analytes and Patient Functional Outcome (55 studies), Neurophysiologic Measures (59 studies), and Tissue Outcome (four studies). In broad terms, this systematic review replicates the pragmatic summary of literature that underpinned the previous consensus statements on CMD [41, 49, 50]. Our analysis strengthens the evidence for many of these associations by identifying a significantly larger number of studies than were cited in recent consensus statements [49, 50]. Further, we document the level of evidence based on conventional evaluation frameworks for evidence based medicine. This assessment found the following evidence:

1. *Patient Functional Outcome -* GRADE level C evidence to support an association between CMD measured glucose, glutamate, glycerol, lactate, LPR and patient functional outcome as assessed by mortality and GOS at 3–6 months post-injury. These CMD measures have over ten studies in support of this relationship, with the majority documenting overwhelming statistical significance. Furthermore, there exists GRADE D level of evidence identified to support a relationship (positive or “nil”) between CMD measured pyruvate, potassium, sodium, and patient outcome. These CMD measures had much less robust literature to support an association with outcome.
2. *Neurophysiologic Measures –* GRADE C evidence for a relationship between elevated LPR and elevated ICP and/or low CPP. We also judged that there was GRADE C evidence supporting an association between elevated glutamate and glycerol with elevated ICP and/or low CPP, and between elevated mean LPR and low PbtO_2_. All remaining CMD measures displayed either GRADE D evidence, or occasionally no evidence, to support a relationship with physiologic measures.
3. *Tissue Outcome –* GRADE D of evidence to suggest an association between LPR in the acute/subacute phase of illness and frontal lobe atrophy at 6 months. The available literature reports no consistent association between CMD measures and MRS or ADC can be made at this time, other than the literature is “unclear”.

However, we had also hoped that integration of the results from these studies would allow us to draw more robust conclusions regarding the relationship of CMD variables and clinical outcome or physiological metrics, with the large summed sample size allowing greater methodological rigor and increased statistical power. However, when compared to large individual studies in the systematic review, the integration of patients from all of the relevant studies did not provide more robust statistical foundations for a specific CPP or ICP threshold. Finally, we found a paucity of data in the area of tissue outcome studies, which was surprising given that follow-up imaging in this population should be common place, and retrospective analysis of CMD measures and imaging based tissue outcome should be possible. We were also surprised that for the tissue outcome studies, the exclusive focus of the CMD measures was lactate, pyruvate and LPR (i.e., markers of metabolic “crisis”). Glutamate and other excitatory amino acids, and markers of tissue distress (glycerol) were not included in most studies on tissue outcome assessment. Inclusion of these markers may display a correlation to acute/subacute and chronic tissue changes.

### Limitations

Despite the interesting results generated by the systematic review, we were unable to grade this evidence as being of a high quality based on RTI criteria, because of the omission of information which might have allowed us to grade the studies at a higher level of evidence. The reasons for this discordance are worth exploring, since its drivers will allow us to help design data collection in a future generation of studies.

One critical set of limitations in most studies was the absence of clear statements that documented the absence of bias in reporting, and hence resulted in categorizing these studies as being at high risk of bias based on the criteria in the RTI item bank [102]. There was some heterogeneity in reporting and some studies may not suffered from the relevant biases, but we were unable to exclude these based on the information provided. One key issue is the basis for selection of the patients included in analysis in individual studies, since this impacts substantially on the generalizability of the conclusions we draw, and the population of patients in whom the results of the study might apply.

A second key issue was the absence of documented blinding of outcome assessors to the CMD results. While the outcome assessors may not have been biased by knowledge of the CMD results, the absence of clear documentation of such blinding results in a downgrading of the quality of evidence in studies based on RTI criteria. In addition, reported thresholds for individual CMD measures and their association with outcome were substantially varied. The definition of metabolic abnormality in previous expert statements drew on knowledge of pathophysiology and of study quality to make pragmatic recommendations regarding metabolic thresholds. This more comprehensive review of the literature shows substantial variability, and without the filter of expert interpretation, does not allow identification of consistent thresholds for most of the measured substrates. Finally, complications (or the absence of complications, and the method by which these were recorded) were seldom reported. This is an issue, an issue since the overall number of patients was 2786 and only four patients were reported as having issues secondary to CMD monitoring, leading to concerns about selective harms reporting. In order to undertake a rigorous analysis of complications/harms associated with CMD catheter placement, we also sought evidence for these secondary endpoints in analysis of publications which reported on physiological or imaging endpoints, but provided no data on clinical outcomes. The available evidence suggests that the placement of CMD probes may well be a low-risk procedure [41, 49, 50], and is accurately reflected by these numbers, but it is impossible to confirm or refute this impression given the available information.

The third critical limitation stems from the focal nature of CMD. Given that the CMD probe only samples a small volume, localized to the tip of the catheter, the results derived from sample analysis provide only insights into focal regional biochemistry. These focal biochemical profiles may not reflect wider, or a more generalized average of, cerebral metabolism. Consequently, inferring relationships between focal CMD biochemistry and global outcomes of interest (functional outcomes, neurophysiology, and/or tissue fate), has significant limitations. However, given the limited ability to accurately measure parenchymal biochemistry and metabolism, we are left with the currently outlined literature base to assess the association between these analytes of interest and various pertinent patient oriented outcomes.

A fourth critical set of limitations was the lack of characterization of patients, especially in many smaller studies. This both contributed to and complicated the limited analysis of differences in CMD findings (and hence outcome associations) in different types of TBI (e.g., diffuse vs. extra-axial hematomas vs. contusions). The location of catheter position and the protocols for CMD sample pooling were not explicitly stated in many studies, and hence make assessment of relationships between monitored variables and outcome relatively crude. In addition, the exact volume of tissue monitored by the CMD probe is not clear, but it is unlikely to be representative of entire regional, hemisphere or global chemistry in the setting of TBI and is likely influenced by injury pattern and treatment. The studies identified within this review fail to delineate injury burden/pattern, and do not address the issue of “focality” of CMD. Thus, our overall conclusions are limited, hence the poor GRADE and RTI assessments for each outcome of interest. The availability of details of ICU management would have provided important insights into the relationships that were observed in some studies and not in others, since certain responses to CMD triggers might have impacted outcome while others might not. Further, it is evident that the CMD biochemical pattern varies significantly over time, during the course of disease evolution following TBI. The timing and duration of these abnormal CMD patterns are of importance. It is possible that either very early abnormalities may have import because they dictate the subsequent course of pathophysiology, or that persistent and late emerging abnormalities in the setting of extreme physiologic events (such as refractory ICP) are more related to clinical outcome. However, the current literature identified within this review is not amenable to such temporal profile analysis and thus our comments on such are limited. Such refined analysis of data with knowledge of key variables would substantially add to the richness and reproducibility of any conclusions that we could draw. Indeed, where such data are available, in both small and large studies, they provide important insights into the information provided by CMD [12, 20, 28, 63–65, 84, 93]. However, these data were not consistently available. Such variations, if unbiased, could be seen to replicate the “noise” seen in pragmatic trials and hence not affect the quality of conclusions that are drawn from the data. However, our inability to ensure that this did not result in bias, resulted in a downgrading of the quality of the studies, and was reflected in our overall RTI assessments and GRADE level of evidence associated with each CMD analyte and the outcomes of interest.

Additionally, given that we do not have robust data to suggest/support “normal” values of CMD measured analytes, we cannot make firm comments on the how the “observed” biochemical changes reported within the CMD studies should be interpreted in relation to “normal” human brain values. We unfortunately are limited to commenting on the relationship between CMD analytes in those patients with poor clinical/physiologic progression versus those with good clinical/physiologic progression. It must be acknowledged that those patients with “good” clinical progression in no way represent “normal” CMD analyte values, but merely the values recorded during a desirable clinical or physiologic trajectory. These recorded CMD analyte values are of course subject to the treatments initiated within the ICU. Thus, in order to better understand what we consider “normal” or “acceptable” levels of various CMD analytes in the setting of TBI, large multi-center archived CMD data is required. Only through correlating the trend in these CMD analytes to: injury pattern/burden, clinical course, other multi-modal physiologic monitoring and the treatments implemented, will we be able to more accurately comment on what analyte values are “normal/acceptable” in the setting of TBI, and what values represent secondary deterioration versus treatment effect. Finally, such data sets would potentially allow for more definitive statements regarding the association between common CMD analytes and the outcomes we address in this review.

A fifth critical limitation was the substantial heterogeneity in studies. The included papers varied by study design, prospective versus retrospective nature, number of patients, patient inclusion criteria, ICU-based therapies offered/provided to patients, blinding during outcome assessment, and primary outcome of the studies. Many studies were written with the purpose of describing primary outcomes other than the ones we sought to review, with outcome information for the purpose of this review provided as incidental information. Selective outcome reporting with regards to individual CMD measures and their association to all outcomes of interest was present in most studies, since the focus in many of these studies was to elucidate pathophysiological relationships, rather than seek outcome associations. Thus, the conclusions that can be drawn from such data are limited. Consequently, most CMD measures were deemed to have GRADE C (low), or D (very low), evidence to support of an association. Given these limitations and heterogeneity issues, a meta-analysis could not be performed.

Finally, it is important to acknowledge that the evidence summary and evaluation that we have undertaken relate exclusively to use of CMD as a variable for specific associations (functional outcome, other neurophysiologic measures, and tissue outcome). The more common use of CMD is as a tool for physiological monitoring, to identify downstream consequences of insufficient oxygen and substrate delivery or mitochondrial dysfunction, and trigger changes in management. Indeed, though prognostic thresholds were identified and published based on the data in these papers, in many instances, the primary aim of the relevant studies was to explore pathophysiology and assess response to therapy. Microdialysis monitoring has undoubtedly informed our understanding of the fundamental biology that underpins metabolic failure following TBI, and provided a rational framework for therapy individualization that can be addressed in interventional trials. The current systematic review process does not address these uses of microdialysis. Similarly, the strength of a relationship between a measured CMD variable and functional, or tissue, outcome could be modified by interventions that were triggered by the CMD measurement. The available data did not allow us to explore the modulatory effect of treatment on the relationship between CMD variables and the various outcomes of interest for this review.

### Technical standardization of microdialysis technique

Cerebral microdialysis is a flexible technique, but this leads to an additional complexity in amalgamating data across studies. These issues can be divided into methodological issues, and targeting of tissue at risk. The commonest microdialysis catheters used in clinical practice are the CMA 70 (20-kDa molecular weight cut off (MWCO)) and CMA 71 (100 kDa MWCO) (CMA Microdialysis AB, Sweden). For the metabolic intermediaries discussed in this review, which are of small molecular weight (~200 Da), the effect of catheter MWCO has little impact on the efficiency of recovery of the molecules of interest [38]. This is not true of CMD for protein biomarkers, but a full discussion of the methodological issues, including alternative perfusion fluids, is outside the scope of this review [35]. The flow rate through the microdialysis catheter also has an impact on the extraction efficiency from the brain ECF, but in clinical applications is typically 0.3 μl/min, although much higher flow rates have been used for samples involving rapid sampling in specialized circumstances.

As a focal technique, CMD catheters sample a small region of brain and the location of the tip of the catheter has an impact on the resulting data. In TBI, several approaches have been taken including two catheter studies comparing perilesional and radiologically normal brain on CT, in the same patient [94]. The results from CMD must therefore be interpreted in an individualized fashion based on the position of the microdialysis catheter tip in relation to specific lesions. However, in general, for patients without a focal lesion the catheter is typically inserted into right frontal lobe i.e. non-dominant, non-eloquent cortex, and for focal lesions such as a contusion or subdural hematoma, the catheter should be placed in the brain adjacent (~2 cm) to the focal lesion targeting brain which is ‘at risk’ but not irretrievably damaged.

### Future directions

There is a large body of literature supporting the association between common CMD measures with both patient functional outcomes and neuro-physiologic measures. This data has provided a basis for pragmatic expert recommendations for use of CMD as a clinical monitoring variable. We confirm these relationships, but our formal assessment of the evidence base suggests many improvements that could be incorporated into the reporting of future studies. We believe that addressing additional factors could provide more robust evidence relating CMD variables to outcome.

First, it seems clear that CMD variables will explain only part of the outcome variation in TBI. This predicates several conclusions. It is likely that large patient numbers will be needed to robustly identify key relationships between CMD and outcome, both overall, and also in selective patho-anatomical groups (e.g. diffuse vs. focal injury). A definitive large multicenter study, focused on CMD, could address this issue, and there is a hope that ongoing prospective studies, including CENTER-TBI [52] and TRACK -TBI [106], may be positioned to answer some of these questions. However, CMD is not part of the core data collection in either of these studies, and patients with CMD monitoring will only represent a minority of the ICU patients recruited. Consequently, it is important that we find ways to make use of all of the data that emerges in this area, including those from smaller studies. We would therefore recommend a common data elements approach to reporting in all CMD studies, with specification of catheter site, location in relation to lesions, type of injury (e.g., by Marshall Grade), CMD thresholds used as triggers in clinical management (ideally as specified by expert consensus statements, while recognizing that these may be different from those used as associations for outcome), and clear documentation of outcome.

Second, it would be useful if the other variables that are known to affect outcome are characterized in publications (with a minimum suggested inclusion of variables that form part of the IMPACT CT model [67, 77, 90, 104]. This would allow us to identify not just the prognostic impact of CMD variable, but also define the supplementary explanatory power that they provide in addition to other well established variables such as GCS and age.

Third, all studies need to explicitly report data that allow assessment of bias. Some of the larger and more rigorous studies in this review explicitly recognized some of these limitations [93], but many neglected to report on these. These data would include the basis on which patients were selected for inclusion, how the study population reflected the broader population of patients in recruiting center, a statement as to whether the outcome assessors were blinded to CMD data, the basis for reporting only some of the CMD variables available, and inclusion of negative results where these are available.

Fourth, to the association between CMD analytes and the three outcome measures we explored needs to be assessed in the context of multimodality monitoring data. Ongoing prospective studies, including CENTER-TBI [52] and TRACK -TBI [106], may be best positioned to answer some of these questions. It is unlikely that a single monitoring technique in isolation will prove sufficient to predict physiology and patient/tissue fate. A combination of multi-modal monitoring techniques will we better enable us to understand the extent of injury and ongoing physiologic/metabolic changes, and arguably stand a better chance of enabling more targeted therapy improved patient outcomes. We would encourage funding bodies to mandate such data collection, and also allow funding provision for such data curation so that legacy research can be of high quality.

Fifth, with regards to imaging-based tissue outcome associations, given the likely availability of data from centers of excellence using CMD in the acute phase, and the common use of follow-up imaging in TBI patients, we believe that a retrospective analysis could substantially refine the way we think about and use CMD, and provide guidance for subsequent prospective studies that address the relationship between CMD variables and tissue fate. It is likely, however, that the patient numbers with both acute CMD data and late imaging are likely to be a minority of the population recruited to research studies, even in centers of excellence. Consequently, a collaborative analysis of the data across centers is likely to be needed to deliver adequate statistical power. Such an analysis will have to address clear pitfalls, including the amalgamation of imaging data from multiple platforms, better characterization of individual patients in each study to allow for covariate adjustment in combined analysis, and innovative statistical approaches to account for late imaging at variable intervals post-TBI to maximize the number of patients who can be included. We also need to reach consensus on the CMD variables that would be included in such analyses, but given the retrospective, hypotheses setting nature of this exercise, a case can be made for attempting to include the widest range of CMD variables.

Finally, it would be useful to consider what imaging modalities would provide the best assessment of tissue fate in such analysis in prospective studies, and when such tissue fate should be assessed following TBI. The choice of imaging modality is difficult. CT is a poor metric of tissue fate in TBI, but its common (near universal) availability could make it attractive as a measure of global or regional atrophy. However, if being undertaken solely for the purpose of such a research analysis, conventional structural MRI has many advantages, including lack of radiation burden, better parenchymal contrast, the ability to differentiate cortical and white matter loss, and good protocols for combining data from different platforms (partly derived from the dementia imaging literature), especially if control data are also available for individual centers. In fact, it would be possible to relate CMD variables to age calibrated metrics of neuronal loss. Indeed, for the next generation of analyses, many of which will be retrospective, a simple T1 weighted image could provide an excellent basic assessment. However, future studies need to take account of the fact that tissue injury in TBI may not present just as pan-necrosis, but also as selective neuronal loss, which may be undetectable by conventional MRI. Detection of this process may provide a more sensitive outcome measure of the metabolic abnormalities detected by CMD on one hand, and a more relevant substrate for functional outcome on the other. Selective neuronal loss (SNL) can be detected by diffusion tensor imaging, MRS or [^11^C] flumazenil PET. The first two of these modalities, and DTI in particular, are rapidly gaining currency as routine research imaging techniques, and show promise for future studies in this context. Further to this, the timing of imaging studies may be crucial in achieving maximal sensitivity for imaging to provide a downstream marker of CMD detected physiological stress. While imaging at 6 months is common, a case can be made for earlier imaging assessment in studies that specifically relationships with acute CMD in the first few days and weeks post TBI, since this could avoid the confound provided by late tissue loss caused by later, and more persistent processes such as neuroinflammation. However, we also need to allow time for any imaging changes to evolve. The retrospective analysis that was discussed earlier, and data from ongoing large prospective studies, such as CENTER-TBI [52] and TRACK-TBI [106], which incorporate serial imaging, could provide guidance on this in the future, but a time interval of 2–3 months might provide the best balance between allowing maturation of the impact of acute physiology and minimizing the confound cause by subacute events.

The more rigorous process of data collection and analysis that we describe will allow use to base future inferences on high-quality studies (even if involving smaller numbers of patients), and better inform the identification of key physiological thresholds related to patient functional outcome and imaging based tissue outcomes. Further studies and analyses need to evaluate the link between abnormal CMD monitoring and other multi-modal monitoring parameters, and to subacute/chronic tissue outcomes. Our goal was to outline the literature, comprehensively, and be critical of the rigor and evidence. It is true that the current CMD literature suffers from limitations, leading to some potential uncertainty regarding its clinical application. Not addressing these issues will restrict the quality of inferences we draw from data.

## Conclusions

There currently exists GRADE C evidence to support an association between low CMD measured glucose and poor patient functional outcome at 3–6 months in severe TBI. Similarly, there exists GRADE C evidence to support an association between high CMD measured glutamate, glycerol, lactate, LPR and poor patient functional outcome at 3–6 months in severe TBI. Furthermore, there currently exists GRADE C level of evidence supporting an association between CMD measured LPR, glutamate and glycerol with ICP and/or CPP. In addition, there currently exists GRADE C evidence to support an association between LPR and PbtO2. All remaining CMD measures displayed either GRADE D evidence, or occasionally no evidence, to support a relationship with physiologic measures. Finally, there currently exists GRADE D evidence to suggest a potential association between elevated CMD measured LPR and frontal lobe atrophy at 6 months post-severe TBI.

All studies reviewed suffer from significant bias, primarily attributable to the primary focus of the individual studies included in this review, which was different from the aims that we sought to explore. Our analysis does not address the use of CMD for characterizing pathophysiology or titrating therapy, and cannot account for any modulation of CMD-outcome relationships by therapy. However they clearly define a role for a new generation of studies analyzing the relationship between common CMD measures and other methods of multi-modal monitoring with patient functional outcome. These need to include the technical specification recommended in past expert guidelines and provide appropriate documentation that allows exclusion of bias.

## Electronic supplementary material

Appendix A (DOC 452 kb)

Appendix B (DOC 225 kb)

Appendix C (DOC 25 kb)

Appendix D (DOC 26 kb)

Appendix E (DOC 119 kb)

Appendix F (DOC 131 kb)

Appendix G (DOC 34 kb)

Appendix H (DOC 27 kb)
