# Supplementary material for: A systematic review of cerebral microdialysis and outcomes in TBI: relationships to patient functional outcome, neurophysiologic measures, and tissue outcome
Source: Acta Neurochir (Wien). 2017 Oct 7;159(12):2245–73. doi: 10.1007/s00701-017-3338-2 (PMC5686263; doi:10.1007/s00701-017-3338-2)

**Appendix A: Sample MEDLINE Search Strategy**

1. TBI.mp. [mp=ti, ab, hw, tn, ot, dm, mf, dv, kw, bt, id, cc, nm, kf, px, rx, an, ui]

2. TBI.tw.

3. traumatic brain injury.mp. [mp=ti, ab, hw, tn, ot, dm, mf, dv, kw, bt, id, cc, nm, kf, px, rx, an, ui]

4. traumatic brain injury.tw.

5. brain injury.mp. [mp=ti, ab, hw, tn, ot, dm, mf, dv, kw, bt, id, cc, nm, kf, px, rx, an, ui]

6. brain injury.tw.

7. head injury.mp. [mp=ti, ab, hw, tn, ot, dm, mf, dv, kw, bt, id, cc, nm, kf, px, rx, an, ui]

8. head injury.tw.

9. cerebral injury.mp. [mp=ti, ab, hw, tn, ot, dm, mf, dv, kw, bt, id, cc, nm, kf, px, rx, an, ui]

10. cerebral injury.tw.

11. head trauma.mp. [mp=ti, ab, hw, tn, ot, dm, mf, dv, kw, bt, id, cc, nm, kf, px, rx, an, ui]

12. head trauma.tw.

13. brain trauma.mp. [mp=ti, ab, hw, tn, ot, dm, mf, dv, kw, bt, id, cc, nm, kf, px, rx, an, ui]

14. brain trauma.tw.

15. cerebral trauma.mp. [mp=ti, ab, hw, tn, ot, dm, mf, dv, kw, bt, id, cc, nm, kf, px, rx, an, ui]

16. cerebral trauma.tw.

17. cerebral microdialysis.mp. [mp=ti, ab, hw, tn, ot, dm, mf, dv, kw, bt, id, cc, nm, kf, px, rx, an, ui]

18. cerebral microdialysis.tw.

19. microdialysis.mp. [mp=ti, ab, hw, tn, ot, dm, mf, dv, kw, bt, id, cc, nm, kf, px, rx, an, ui]

20. microdialysis.tw.

21. dialysis. [mp=ti, ab, hw, tn, ot, dm, mf, dv, kw, bt, id, cc, nm, kf, px, rx, an, ui]

22. dialysis.tw.

23. MDialysis.mp. [mp=ti, ab, hw, tn, ot, dm, mf, dv, kw, bt, id, cc, nm, kf, px, rx, an, ui]

24. MDialysis.tw.

25. MD probe.mp. [mp=ti, ab, hw, tn, ot, dm, mf, dv, kw, bt, id, cc, nm, kf, px, rx, an, ui]

26. MD probe.tw.

27. microdialysate.mp. [mp=ti, ab, hw, tn, ot, dm, mf, dv, kw, bt, id, cc, nm, kf, px, rx, an, ui]

28. microdialysate.tw.

29. dialysate.mp. [mp=ti, ab, hw, tn, ot, dm, mf, dv, kw, bt, id, cc, nm, kf, px, rx, an, ui]

30. dialysate.tw.

31. lactate.mp. [mp=ti, ab, hw, tn, ot, dm, mf, dv, kw, bt, id, cc, nm, kf, px, rx, an, ui]

32. lactate.tw.

33. pyruvate.mp. [mp=ti, ab, hw, tn, ot, dm, mf, dv, kw, bt, id, cc, nm, kf, px, rx, an, ui]

34. pyruvate.tw.

35. LP ratio.mp [mp=ti, ab, hw, tn, ot, dm, mf, dv, kw, bt, id, cc, nm, kf, px, rx, an, ui]

36. LP ratio.tw

37. LP.mp [mp=ti, ab, hw, tn, ot, dm, mf, dv, kw, bt, id, cc, nm, kf, px, rx, an, ui]

38. LP.tw

39. lactic acid.mp [mp=ti, ab, hw, tn, ot, dm, mf, dv, kw, bt, id, cc, nm, kf, px, rx, an, ui]

40. lactic acid.tw

41. glutamate.mp [mp=ti, ab, hw, tn, ot, dm, mf, dv, kw, bt, id, cc, nm, kf, px, rx, an, ui]

42. glutamate.tw

43. glutamic acid.mp [mp=ti, ab, hw, tn, ot, dm, mf, dv, kw, bt, id, cc, nm, kf, px, rx, an, ui]

44. glutamic acid.tw

45. glucose.mp [mp=ti, ab, hw, tn, ot, dm, mf, dv, kw, bt, id, cc, nm, kf, px, rx, an, ui]

46. glucose.tw

47. glycerol.mp [mp=ti, ab, hw, tn, ot, dm, mf, dv, kw, bt, id, cc, nm, kf, px, rx, an, ui]

48. glycerol.tw

49. 1 or 2 or 3 or 4 or 5 or 6 or 7 or 8 or 9 or 10 or 11 or 12 or 13 or 14 or 15 or 16

50. 17 or 18 or 19 or 20 or 21 or 22 or 23 or 24 or 25 or 26 or 27 or 28 or 29 or 30

51. 31 or 32 or 33 or 34 or 35 or 36 or 37 or 38 or 39 or 40 or 41 or 42 or 43 or 44 or 45 or 46 or 47 or 48

52. 49 and 50

53. 52 and 51

54. remove duplicates from 53

**Meetings Searched for Following Professional Societies:**

Canadian Neurological Sciences Federation (CNSF), American Association of Neurological Surgeons (AANS), Congress of Neurological Surgeons (CNS), European Neurosurgical Society (ENSS), World Federation of Neurological Surgeons (WFNS), National Neurotrauma Society (NNS), American Neurology Association (ANA), American Academy of Neurology (AAN), European Federation of Neurological Science (EFNS), World Congress of Neurology (WCN), Society of Critical Care Medicine (SCCM), Neurocritical Care Society (NCS), European Society for Intensive Care Medicine (ESICM), World Federation of Societies of Intensive and Critical Care Medicine (WFSICCM), American Society for Anesthesiologists (ASA), World Federation of Societies of Anesthesiologist (WFSA), Australian Society of Anesthesiologists, International Anesthesia Research Society (IARS), Society of Neurosurgical Anesthesiology and Critical Care (SNACC), Society for Neuroscience in Anesthesiology and Critical Care, Japanese Society of Neuroanesthesia and Critical Care (JSNCC), International NeuroTrauma Society (INTS), International Brain Injury Association (IBIA), and the College of Intensive Care Medicine Annual Scientific Meeting (CICMASM - Australia).

PRISMA Flow Diagrams

**Figure 1: Flow Diagram of Search Results – Functional Outcome Studies**

**
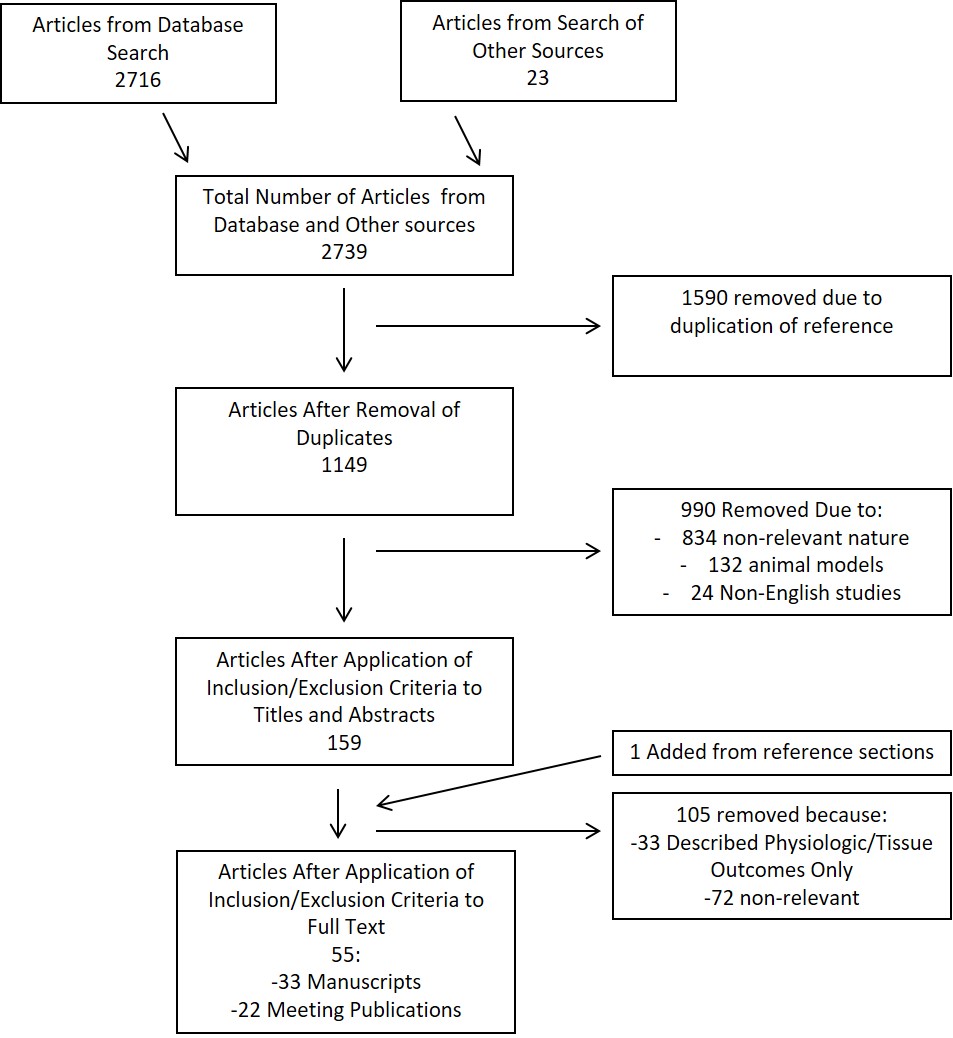
**

**Figure 2: Flow Diagram of Search Results – Neurophysiologic Measure Studies**

**
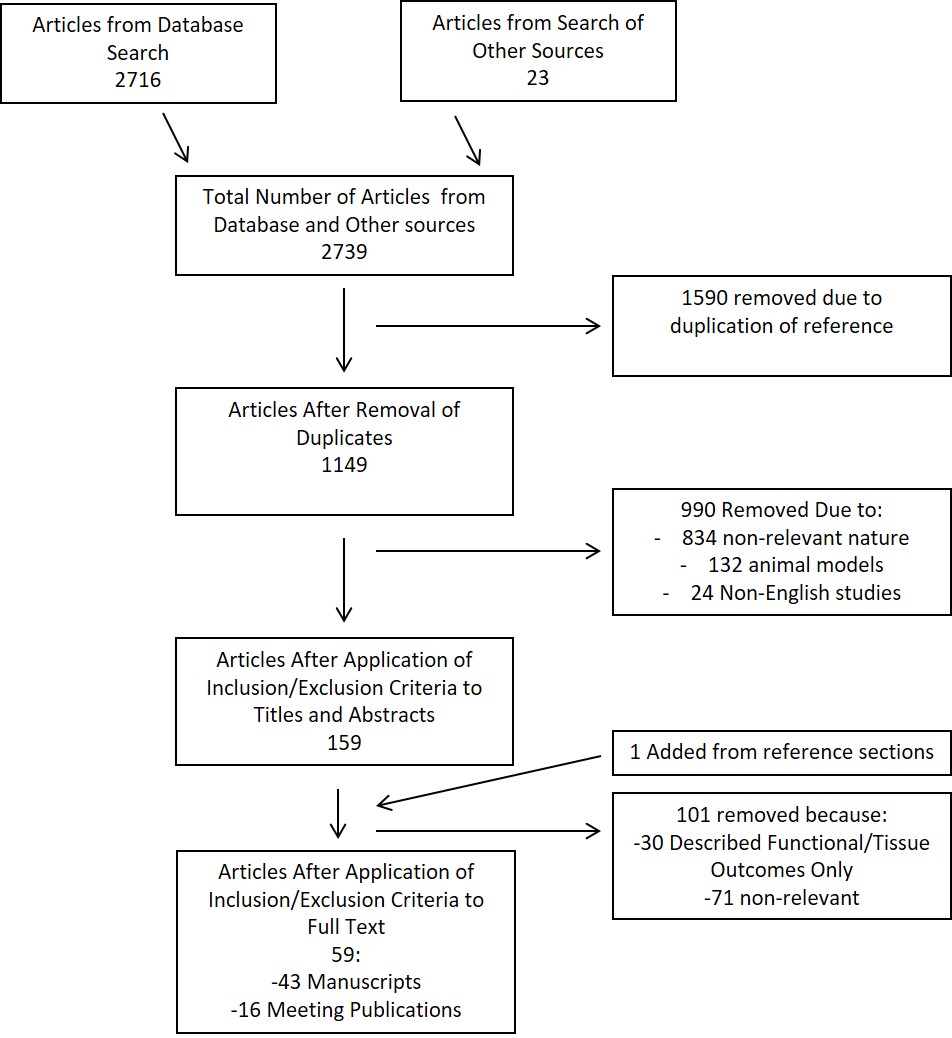
**

**Figure 3: Flow Diagram of Search Results – Tissue Outcome Studies**


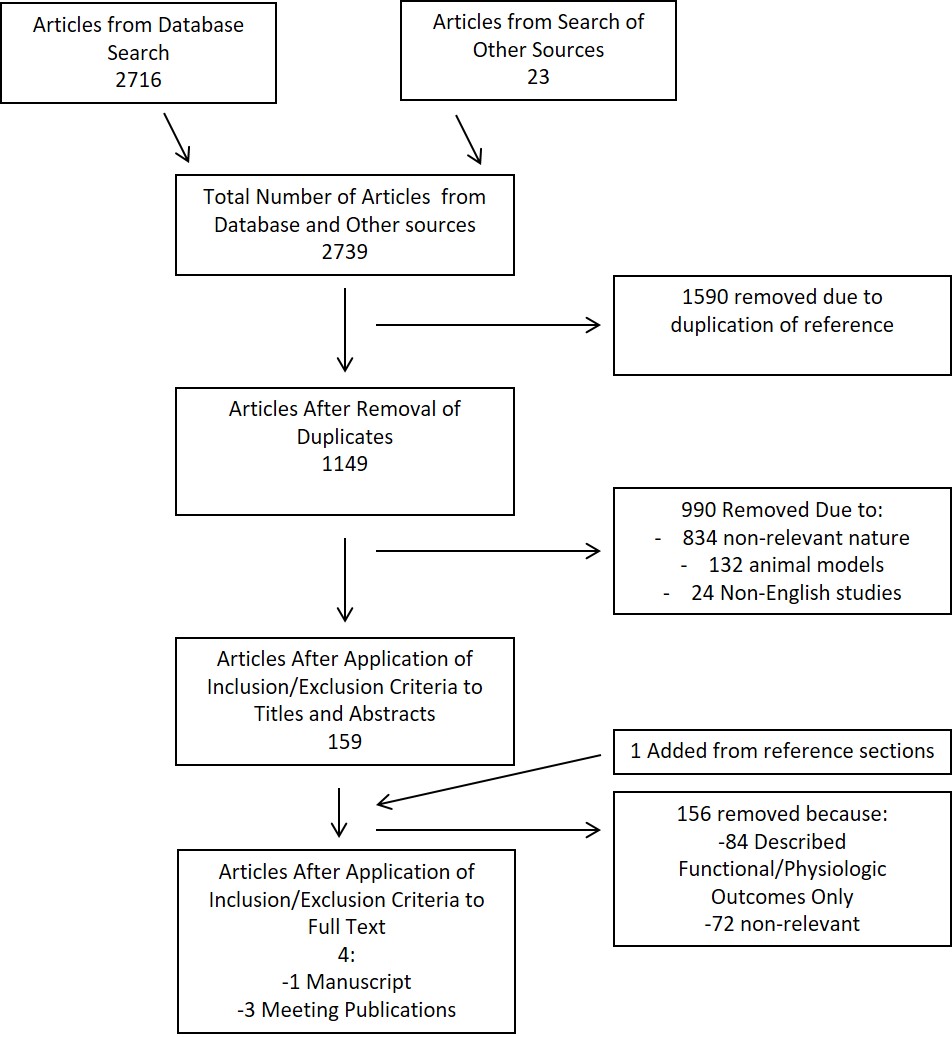

Supplement: Supplementary file 1 — (DOC 452 kb) [file 701_2017_3338_MOESM1_ESM.doc]
