# Supplementary material for: A systematic review of cerebral microdialysis and outcomes in TBI: relationships to patient functional outcome, neurophysiologic measures, and tissue outcome
Source: Acta Neurochir (Wien). 2017 Oct 7;159(12):2245–73. doi: 10.1007/s00701-017-3338-2 (PMC5686263; doi:10.1007/s00701-017-3338-2)
Supplement: Supplementary file 2 — (DOC 225 kb) [file 701_2017_3338_MOESM2_ESM.doc]

Appendix B – RTI Item Bank Bias Tables

Bias Tables – Functional Outcome Studies

| Reference | Inclusion/Exclusion Criteria | Comparison Group | Recruiting | Performance Bias | Blinding | Valid Measures of Outcome | Follow Up Length | Attrition Assessment | Selective Outcome Reporting | Harms Reporting | Confounders Considered | Overall Bias Assessment |
| --- | --- | --- | --- | --- | --- | --- | --- | --- | --- | --- | --- | --- |
| **Positive Association Studies** | | | | | | | | | | | | |
| Alessandri et al [2] | U | H | U | H | H | L | U | U | H | H | H | H |
| Badenes et al [6] | U | L | U | U | H | L | U | U | H | H | H | H |
| Belli et al [8] | L | H | L | H | H | L | L | U | L | L | H | H |
| Bidot et al [9] | U | H | U | U | H | L | H | H | H | U | H | H |
| Bolcha et al [10] | U | H | U | U | H | L | L | U | H | H | H | H |
| Bullock et al [12] | H | H | L | H | H | L | U | U | L | L | H | H |
| Chamoun et al [13] | L | H | L | H | H | L | L | U | L | L | H | H |
| Chan et al [14] | U | H | U | H | H | U | U | U | H | H | H | H |
| Clausen et al [17] | U | H | H | H | H | L | L | U | H | H | H | H |
| Clausen et al [18] | H | H | H | H | H | L | L | U | H | H | H | H |
| Dizdarevic et al [20] | L | L | L | H | H | L | U | U | H | H | H | H |
| Figaji et al [21] | H | H | U | H | H | U | U | U | H | H | H | H |
| Goodman et al [25] | L | H | H | H | H | U | U | H | L | H | H | H |
| Gopinath et al [27] | H | H | U | H | H | L | U | U | H | H | H | H |
| Gupta et al [29] | L | H | H | H | H | L | L | H | H | H | H | H |
| Gupta et al [28] | L | H | U | H | H | L | U | U | H | H | H | H |
| Hejcl et al [34] | H | H | H | H | H | U | L | U | H | H | H | H |
| Hutchinson et al [38] | H | H | U | H | H | U | H | U | H | H | H | H |
| Igarashi et al [42] | H | H | U | H | H | L | U | U | H | H | H | H |
| Johnston et al [44] | L | H | L | L | H | U | U | U | H | H | H | H |
| Karathanou et al [45] | H | H | U | H | H | L | L | U | H | H | H | H |
| Koura et al [47] | H | H | L | H | H | U | U | U | H | H | H | H |
| Kurtz et al [48] | H | H | H | H | H | L | H | H | H | H | H | H |
| Li et al [51] | H | H | H | H | H | L | H | H | H | H | H | H |
| Marcoux et al [53] | L | H | H | H | H | L | L | H | H | H | H | H |
| Mazzeo et al [54] | H | H | H | H | H | L | L | H | H | H | H | H |
| Mellergard et al [55] | L | H | H | L | H | H | L | H | H | H | H | H |
| Nordstrom et al [62] | L | H | H | L | H | H | L | H | H | H | H | H |
| Oddo et al [64] | H | H | H | H | H | H | H | H | H | H | H | H |
| Olivecrona et al [65] | L | H | H | L | H | H | L | H | H | L | H | H |
| Omerhodzic et al [66] | H | H | H | H | H | U | U | U | H | H | H | H |
| Paraforou et al [69] | H | H | H | L | H | L | L | H | H | H | H | H |
| Peerdeman et al [70] | L | H | L | H | H | L | L | H | H | H | H | H |
| Petzold et al [71] | L | H | L | H | H | L | L | H | H | H | H | H |
| Reinert et al [73] | U | H | H | H | H | L | L | H | H | H | H | H |
| Reinert et al [74] | L | H | H | H | H | L | L | H | H | H | H | H |
| Richards et al [75] | L | H | L | H | H | L | L | H | H | H | H | H |
| Robertson et al [76] | U | H | U | H | H | U | U | U | H | H | H | H |
| Sanchez-Porras et al [81] | H | H | H | H | H | U | U | H | H | H | H | H |
| Sanchez et al [80] | U | H | U | H | H | L | H | U | H | H | H | H |
| Singla et al [86] | H | H | H | H | H | L | L | H | H | H | H | H |
| Stein et al [88] | L | H | L | H | H | L | L | H | H | H | H | H |
| Stiefel et al [91] | H | H | H | H | H | U | U | U | H | H | H | H |
| Timofeev et al [93] | H | H | H | H | H | L | L | H | H | H | H | H |
| Vespa et al [99] | L | H | L | H | H | L | L | H | H | L | H | H |
| Wang et al [103] | U | H | U | H | H | L | U | U | H | H | H | H |
| Yokobori et al [105] | L | H | L | H | H | L | U | H | H | H | H | H |
| Zauner et al [107] | U | H | U | H | H | U | U | U | H | H | H | H |
| Zauner et al [108] | H | H | H | H | H | L | L | U | H | H | H | H |
| **Nil Association Studies** | | | | | | | | | | | | |
| Alessandri et al [3] | U | H | U | H | H | U | U | U | H | H | H | H |
| Chen et al [16] | H | H | H | H | H | U | U | U | H | H | H | H |
| Nelson et al [60] | H | H | H | H | H | L | L | H | H | H | H | H |
| Nelson et al [61] | H | H | H | H | H | L | L | H | H | H | H | H |
| Papanikolaou et al [68] | U | H | U | H | H | U | U | U | H | H | H | H |
| Thelin et al [92] | H | H | H | H | H | L | L | H | H | H | H | H |

Bias Tables – Neuro-physiologic Measure Studies

| Reference | Inclusion/Exclusion Criteria | Comparison Group | Recruiting | Performance Bias | Blinding | Valid Measures of Outcome | Follow Up Length | Attrition Assessment | Selective Outcome Reporting | Harms Reporting | Confounders Considered | Overall Bias Assessment |
| --- | --- | --- | --- | --- | --- | --- | --- | --- | --- | --- | --- | --- |
| **ICP/CPP Positive Association Studies** | | | | | | | | | | | | |
| Adamides et al [1] | L | H | L | L | H | L | H | U | H | H | H | H |
| Belli et al [7] | L | H | L | H | H | L | H | U | H | L | H | H |
| Bolcha et al [10] | U | H | U | U | H | L | L | U | H | H | H | H |
| Bullock et al [12] | H | H | L | H | H | L | U | U | L | L | H | H |
| Clausen et al [17] | U | H | H | H | H | L | L | U | H | H | H | H |
| Clausen et al [18] | H | H | H | H | H | L | L | U | H | H | H | H |
| Goodman et al [26] | H | H | H | H | H | L | H | H | H | H | H | H |
| Goodman et al [25] | L | H | H | H | H | U | U | H | L | H | H | H |
| Gupta et al [28] | L | H | U | H | H | L | U | U | H | H | H | H |
| Hejcl et al [34] | H | H | H | H | H | U | L | U | H | H | H | H |
| Koura et al [47] | H | H | L | H | H | U | U | U | H | H | H | H |
| Kurtz et al [48] | H | H | H | H | H | L | H | H | H | H | H | H |
| Li et al [51] | H | H | H | H | H | L | H | H | H | H | H | H |
| Meixensberger et al [56] | H | H | H | H | H | L | H | H | H | H | H | H |
| Nelson et al [61] | H | H | H | H | H | L | L | H | H | H | H | H |
| Nordstrom et al [62] | L | H | H | L | H | H | L | H | H | H | H | H |
| Papanikolaou et al [68] | U | H | U | H | H | U | U | U | H | H | H | H |
| Paraforou et al [69] | H | H | H | L | H | L | L | H | H | H | H | H |
| Richards et al [75] | L | H | L | H | H | L | L | H | H | H | H | H |
| Salci et al [79] | H | H | H | H | H | L | H | H | H | H | H | H |
| Sarrafzadeh et al [82] | U | H | U | H | H | H | H | U | H | H | H | H |
| Singla et al [86] | H | H | H | H | H | L | L | H | H | H | H | H |
| Stahl et al [87] | H | H | H | H | H | H | H | H | H | H | H | H |
| Stein et al [89] | U | H | U | H | H | H | U | H | H | H | H | H |
| Stiefel et al [91] | H | H | H | H | H | U | U | U | H | H | H | H |
| Timofeev et al [93] | H | H | H | H | H | L | L | H | H | H | H | H |
| Timofeev et al [94] | U | H | U | H | H | L | U | U | H | H | H | H |
| Vespa et al [98] | L | H | L | H | H | H | H | U | H | H | H | H |
| ***PbtO2/SjvO2 Positive Association Studies*** | | | | | | | | | | | | |
| Chan et al [15] | U | H | U | H | H | U | U | U | H | H | H | H |
| Figaji et al [21] | H | H | U | H | H | U | U | U | H | H | H | H |
| Menzel et al [57] | H | H | H | H | H | L | L | U | H | H | H | H |
| Menzel et al [58] | L | H | H | H | H | H | H | U | H | H | H | H |
| Nortje et al [63] | L | H | L | L | H | L | L | L | L | H | H | H |
| Purins et al [72] | L | H | L | H | H | L | L | L | L | H | H | H |
| Robertson et al [76] | U | H | U | H | H | U | U | U | H | H | H | H |
| Sarrafzadeh et al [83] | H | H | H | H | H | L | L | L | H | H | H | H |
| Sarrafzadeh et al [84] | U | H | U | H | H | L | L | U | H | H | H | H |
| Timofeev et al [95] | L | H | L | H | H | L | L | U | H | H | H | H |
| Valdaka et al [96] | H | H | H | H | H | H | H | H | H | H | H | H |
| Vilalta et al [101] | L | H | L | H | H | L | L | L | H | H | H | H |
| ***Autoregulation - Positive Association Studies*** | | | | | | | | | | | | |
| Asgari et al [4] | H | H | H | H | H | U | U | H | H | H | H | H |
| Asgari et al [5] | L | H | L | H | H | U | U | H | H | H | H | H |
| Yokobori et al 105] | L | H | L | H | H | L | U | H | H | H | H | H |
| ***Imaging Based Positive Association Studies*** | | | | | | | | | | | | |
| Bouzat et al [11] | L | H | L | H | H | L | L | H | H | H | H | H |
| Hutchinson et al [39] | L | H | L | L | H | L | L | H | H | H | H | H |
| Hutchinson et al [40] | L | H | L | L | H | L | L | H | H | H | H | H |
| Reinert et al [74] | L | H | H | H | H | L | L | H | H | H | H | H |
| Sala et al [78] | L | H | L | L | H | L | L | H | H | H | H | H |
| Vespa et al [97] | L | H | L | L | H | L | L | H | H | H | H | H |
| Vespa et al [99] | L | H | L | H | H | L | L | H | H | L | H | H |
| Zauner et al [109] | U | H | U | H | H | L | L | U | H | H | H | H |
| **Negative Association Studies** | | | | | | | | | | | | |
| Alessandri et al [3] | U | H | U | H | H | U | U | U | H | H | H | H |
| Chamoun et al [13] | L | H | L | H | H | L | L | U | L | L | H | H |
| Johnston et al [44] | L | H | L | L | H | U | U | U | H | H | H | H |
| Nelson et al [60] | H | H | H | H | H | L | L | H | H | H | H | H |
| Peerdeman et al [70] | L | H | L | H | H | L | L | H | H | H | H | H |
| Petzold et al [71] | L | H | L | H | H | L | L | H | H | H | H | H |
| Sanchez-Porras et al [81] | H | H | H | H | H | U | U | H | H | H | H | H |
| Vespa et al [100] | L | H | L | H | H | L | L | H | H | H | H | H |

Bias Tables – Tissue Outcome Studies

| Reference | Inclusion/Exclusion Criteria | Comparison Group | Recruiting | Performance Bias | Blinding | Valid Measures of Outcome | Follow Up Length | Attrition Assessment | Selective Outcome Reporting | Harms Reporting | Confounders Considered | Overall Bias Assessment |
| --- | --- | --- | --- | --- | --- | --- | --- | --- | --- | --- | --- | --- |
| **Positive Association Studies** | | | | | | | | | | | | |
| Filippou et al [23] | U | H | U | H | H | U | U | U | H | H | H | H |
| Marcoux et al [53] | L | H | H | H | H | L | L | H | H | H | H | H |
| **Nil Association Studies** | | | | | | | | | | | | |
| Filippou et al [22] | U | H | U | H | H | U | U | U | H | H | H | H |
| Stein et al [89] | U | H | U | H | H | U | U | U | H | H | H | H |

*Summary*

The formally published manuscripts were all deemed high-risk overall, with some variation in degree of risk for individual categories. The main high risk features for the manuscripts included: poorly specified inclusion criteria with severe TBI patients of “all types”, no stratification of CMD/functional/neuro-physiologic/tissue outcome measures based on injury patterns, procedural bias based on timing of CMD measures and locations of catheters relative to injury/healthy tissue, lack of blinding, selective outcome reporting in many (given no harms records and partial reporting of CMD measures), and high risk of confounding variables not specified (for example, significant heterogeneity in ICU treatment).

The 3 RCTs included within the functional outcome aspect of this review were also deemed high risk overall for bias. The defined primary outcome of our review did not match the primary outcome within these studies. The data for CMD measure relations to functional outcome was selectively reported within these papers, given that is was not the main focus of the study design. Hence, it was necessary to rank these studies as “high-risk” for the purpose of this systematic review. Furthermore, these studies may have suffered from patient selection bias, lack of blinding, and were at risk for confounders (such as variation in individual patient treatment and comorbidities).

*Factors Considered in Bias Assessment for RTI Item Bank*

1. Inclusion/Exclusion criteria – Studies were assessed on the extent of their listed criteria. Bias was considered to be present if studies failed to consider the complexity of TBI included (injury patterns) as this could easily influence results. Not considering pre-hospital or emergency department based episodes of secondary injury was considered a flaw in the inclusion/exclusion criteria. Including wide age ranges and all mechanisms of injury was a source of bias.
2. Comparison Group – lack of comparison groups was considered a bias, poorly delineated comparison groups with inter-group variation in demographics was considered a bias.
3. Recruiting – retrospective database mining was considered high risk. Poorly described methods of patient recruitment/consent were considered biased. Undefined periods of recruitment and small sample size were considered a form of bias.
4. Performance bias – microdialysis monitoring techniques that were vaguely described; lack of information on tissue location, and sample collection were considered biased. Similarly, if varied time frames of physiologic assessment were utilized (or time frame to measurement wasn’t mentioned/specified), without specified standardized techniques, this was considered a source of bias.
5. Blinding – lack of blinding of data to outcome measure assessment was considered a source of bias.
6. Valid Outcome Measures – we defined methods of neuro-physiologic measurement which were considered low bias risk. Studies reporting novel techniques raised questions of validity of outcome measure and were thus considered potentially biased.
7. Duration of Follow-up – if not defined or poorly documented follow-up was found, these studies were considered high bias.
8. Attrition – lack of comments regarding delays in physiologic or CMD measurements, or missing data points for measures, were considered for potential bias in the consideration of attrition.
9. Selective Outcome Reporting – lack of reporting association between all measured CMD substrates and physiologic parameters was considered high risk of bias.
10. Harms Reporting – lack of complication documentation within formal manuscripts was considered selective harms reporting bias. Meeting abstracts were graded as “unclear” given the limitation in reporting available for abstracts.
11. Confounders – if sources of secondary brain injury, variation in hospital/ICU treatment and lack of consideration for injury patterns and CMD techniques weren’t considered then studies were considered biased.
12. Overall Assessment – based on consensus between two reviewers and their overall impression of the bias assessment, study design, and limitations.
