# Supplementary material for: A systematic review of cerebral microdialysis and outcomes in TBI: relationships to patient functional outcome, neurophysiologic measures, and tissue outcome
Source: Acta Neurochir (Wien). 2017 Oct 7;159(12):2245–73. doi: 10.1007/s00701-017-3338-2 (PMC5686263; doi:10.1007/s00701-017-3338-2)
Supplement: Supplementary file 3 — (DOC 25 kb) [file 701_2017_3338_MOESM3_ESM.doc]

**Appendix C: CMD Measurement Techniques Described**

1. *Functional Outcome Studies*

Location of the CMD probe was documented in 29 studies as: healthy tissue in 12 studies, [8,17,18,44,53,54,70,75,88,99,107,108] Peri-lesional in 5 studies, [20,29,69,92,105] and mixed placement within the cohort in 12 studies. [12,25,51,60-62,64-66,71,74,93] The remaining studies failed to clarify the tissue location (healthy, peri-lesional, or mixed) in which the CMD catheter was located.

1. *Neuro-physiologic Measure Studies*

The details surrounding CMD analytes and technique were available in varying detail in most included studies. Probe tissue location (i.e. structurally normal-appearing, peri-lesional, or mixed) was mentioned in 27 studies, with the following reported locations: structurally normal-appearing/healthy tissue in 10 studies, [11,17,18,26,70,71,75,82,99,101] peri-lesional tissue in 4 studies, [7,56,69,87] and mixed tissue location in 13 studies. [1,12,25,51,60-62,71,74,87,89,95,100] Unfortunately, 32 studies failed to clarify the tissue location of the CMD probe.

Measured CMD analytes typically consisted of a panel (glucose, lactate, pyruvate, LPR, +/-glutamate). Some studies focused on only 1 CMD measure, such as glutamate or potassium. Reporting of CMD measures in relation to physiologic outcome was highly selective within many studies.

The most commonly reported interval of CMD measure was hourly. A minority of studies reported more frequent analysis. Unfortunately, most studies were unclear as to whether the CMD samples collected were analyzed during those time frames described for collection, or pooled for analysis based on specific time periods.

1. *Tissue Outcome Studies*

All studies measured lactate, pyruvate, and LPR. Glucose was measured in 3 studies [23,53,89] and glutamate in 1 study. [53] Only 1 study reported a clear tissue location for the CMD probe, where it was intentionally placed in healthy tissue. The interval for CMD collection was also incompletely documented in the studies, with only one study mentioning “hourly collection”. [53] The associations were with summary data averaged over several hours or time spent above a set threshold.
