# Supplementary material for: A systematic review of cerebral microdialysis and outcomes in TBI: relationships to patient functional outcome, neurophysiologic measures, and tissue outcome
Source: Acta Neurochir (Wien). 2017 Oct 7;159(12):2245–73. doi: 10.1007/s00701-017-3338-2 (PMC5686263; doi:10.1007/s00701-017-3338-2)
Supplement: Supplementary file 4 — (DOC 26 kb) [file 701_2017_3338_MOESM4_ESM.doc]

**Appendix D: Summary of Outcome Measurement Techniques**

1. *Patient Functional Outcome*

Patient functional outcome was reported in all included studies. The measure of outcome varied significantly across the included studies. Mortality (only) was reported in 9 studies, [6,10,48,51,55,62,64,65,71] with the typical interval of reporting at 6 months post-injury. Glasgow Outcome Scale – Extended (GOSE) was reported in 3 studies at 6 months post-injury. [53,88,99]

The most commonly reported outcome measure was the GOS, with 27 studies utilizing some form of this scale. Within this group of studies, a dichotomized GOS (Good = 4 or 5; Poor = 1 to 3) was reported in 20 studies at intervals ranging from 3 to 6 months post-injury. [2,8,9,12,13,17,18,20,29,45,61,70,73,74,80,81,93,103] A trichotomized Glasgow Outcome Scale (GOS) was reported in one study, at an undefined interval post-injury. [60] Finally, 6 studies reported the use of GOS in a non-partitioned manner. [27,28,54,69,75,86]

Sixteen studies included in the review failed to specify the exact outcome measure that was compared with CMD substrates. [3,14,16,21,25,34,38,42,44,47,66,68,76,91,107,108] These studies typically reported “good” or “poor” outcome, without further explanation of criteria utilized to categorize the patients accordingly.

1. *Neuro-physiologic Measures*

A variety of different techniques were correlated to common CMD measures, with the following main technique described: ICP/CPP in 28 studies, [1,7,10,12,17,18,25,26,28,34,47,48,51,56,61,62,68,69,75,79,82,86,87,89,91,92,94,98] PbtO_2_ in 11 studies, [21,57,58,63,72,76,83,84,95,96,101] SjvO_2_ in 1 study, [15] autoregulation in 3 studies, [4,5,105] and imaging based assessment of cerebral physiology or metabolism (computed tomographic perfusion (CTP), Xenon computed tomography (Xe CT), PET – fluorodeoxy glucose (FDG)/^15^O) in 8 studies. [11,39,40,74,78,97,99,109] Some studies reported associations between multiple physiologic techniques.

Eight studies explored varying associations (mainly of CMD variables with ICP/CPP) to demonstrate the absence of a relationship between CMD and physiologic associations. [3,13,44,60,70,71,81,100] Some studies reporting positive associations between specific CMD measures and physiologic measures also described no associations for other CMD measures.

1. *Tissue Outcome Measures*

All studies measured lactate, pyruvate, and LPR. Glucose was measured in 3 studies [23,53,89] and glutamate in 1 study. [53] Only 1 study reported a clear tissue location for the CMD probe, where it was intentionally placed in healthy tissue. The interval for CMD collection was also incompletely documented in the studies, with only one study mentioning “hourly collection”. [53] The associations were with summary data averaged over several hours or time spent above a set threshold.
