# Supplementary material for: A systematic review of cerebral microdialysis and outcomes in TBI: relationships to patient functional outcome, neurophysiologic measures, and tissue outcome
Source: Acta Neurochir (Wien). 2017 Oct 7;159(12):2245–73. doi: 10.1007/s00701-017-3338-2 (PMC5686263; doi:10.1007/s00701-017-3338-2)
Supplement: Supplementary file 5 — (DOC 119 kb) [file 701_2017_3338_MOESM5_ESM.doc]

Appendix E: CMD Measures and Functional Outcomes

| **Reference** | **Catheter Location and**  **Measured CMD Analytes of Interest** | **Interventional Therapies Applied During Measurement** | **Outcome Measure Technique** | **CMD Analytes and**  **Patient Functional Outcome** | **Complications to CMD** | **Conclusions** |
| --- | --- | --- | --- | --- | --- | --- |
| **Positive Association Studies** | | | | | | |
| Alessandri et al [2] | *Unclear Location*  ***Glutamate and Lactate***  (Unclear Interval) | Not Specified | Dichotomized GOS at unspecified interval (Good = 4 or 5; Poor = 1 to 3) | Glutamate and Lactate elevations after the first 12 hours were associated with Poor outcome | Not Specified | Glutamate/Lactate Elevations after the first 12 hours are Associated to Poor Outcome |
| Badenes et al [6] | *Unclear Location*  ***Glucose***  (Unclear Interval) | RCT of Intensive Glycemic Control  *Group 1 (n=15):* Continuous Insulin Infusion to maintain BG 4-8 mmol/L  *Group 2 (n=15):* BG goal 8.1-10 mmol/L | Mortality at unspecified interval | Low CMD Glucose Linked to Non-survivors (mean 0.46 mmol/L versus 1.04 mmol/L) (p<0.05) | Not Specified | Low CMD Glucose is associated with mortality |
| Belli et al [8] | *Healthy Tissue*  ***Lactate, Pyruvate, Glutamate, Glycerol***  (q12 Hour Measure) | Not Specified | Dichotomized GOS (Good = 4 or 5; Poor = 1 to 3) at 5 to 9 Months | High LPR associated with Non-survivors (p=0.001)  High Glycerol associated with Non-survivors (p=0.0001)  No association between glutamate and outcome | ICH in 1 patient | High LPR and Glycerol Associated with Mortality  Glutamate Not Associated with Mortality |
| Bidot et al [9] | *Unclear Location*  ***Glucose, Lactate, Pyruvate, LPR***  (Unclear Interval) | Not Specified | Dichotomized GOS at discharge (Good >4; Poor<3) | High Glucose (p<0.001), Lactate (p<0.01), Pyruvate, and LPR (p<0.05) Associated with Poor Outcome  Early (day 4) drop in CMD glucose linked to Poor Outcome | Not Specified | High Glucose, Lactate, Pyruvate, and LPR are associated with Poor Outcome at discharge |
| Bolcha et al [10] | *Unclear Location*  ***Glucose, Glycerol, LPR***  (Unclear Interval) | Not Specified | Mortality at 6 months | High LPR and Glycerol was associated with fatal outcome at 6 months | Not Specified | High LPR and Glycerol are associated with fatal outcome |
| Bullock et al [12] | *Mixed Peri-Lesion and Healthy Tissue*  ***Glutamate***  (q30 min Measure for 4 days) | Standard ICP Therapy; “Few” with barbiturate infusions | Dichotomized GOS at unclear interval (Good = 4 or 5; Poor = 3 or less) | High Glutamate (>20 umol/L) associated with poor outcome (p<0.05) | 3 catheters malfunctioned requiring replacement | High Glutamate is associated with Poor Outcome |
| Chamoun et al [13] | *Unclear Location*  ***Glutamate***  (Hourly Measure) | Various ICP Therapies; Some DC patients | Dichotomized GOS at 6 Months (Good = 4 or 5; Poor = 3 or less) | High Glutamate (>20 umol/L) associated with Poor Outcome (p=0.03) | Not Specified | High Glutamate Associated with Poor Outcome at 6 Months |
| Chan et al [14] | *Unclear Location*  ***Glutamate***  (Unclear Interval) | Not Specified | Unspecified Outcome Scale at Unclear Interval | Low Pearson Coefficient (<-0.5) – indicating high Glutamate during low CPP episodes was linked to poor outcome at 6 months (p<0.001) | Not Specified | High Glutamate is Associated with Poor Outcome |
| Clausen et al [17] | *Healthy Tissue*  ***Glycerol***  (q30 min Measure) | Various ICP Therapies | Dichotomized GOS at 3 Months (Good = 4-5; Poor = 1-2) | High mean Glycerol (mean 83 umol/L versus 63 umol/L) level over course of ICU stay was associated with poor outcome (p<0.01) | Not Specified | High Glycerol is associated with Poor Outcome at 3 Months |
| Clausen et al [18] | *Healthy Tissue*  ***Lactate***  (q30 min Measure) | Various ICP Therapies; Barbiturates; Hypothermia | Dichotomized GOS at 3 Months (Good = 4 or 5; Poor = 1 or 2) | High Lactate (mean over stay of 1051 umol/L verus 785 umol/L) is associated with Poor Outcome | Not Specified | High Lactate is Associated with Poor Outcome at 3 Months |
| Dizdarevic et al [20] | *Peri-Lesional*  ***Lactate, LPR, Glycerol***  (q2 Hour Measure) | RCT Comparing Lund Therapy (n=15) vs. CPP Directed Therapy (n=15) | Dichotomized GOS (Good = 4 or 5; Poor = 1 to 3) at Unclear Interval | Low Glucose is Associated with Poor Outcome (mean 0.96 mmol/L versus 1.12 mmol/L) (p=0.003)  Elevated Glycerol is Associated with Poor Outcome (mean 193 umol/L versus 96.5 umol/L) (p=0.02)  Elevated LPR is Associated with Poor Outcome (mean 36.5 versus 20) (p=0.01) | Not Specified | Low Glucose, High Glycerol/LPR are Associated with Poor Outcome |
| Figaji et al [21] | *Unclear Location*  ***Lactate, LPR, glycerol***  (Unclear Interval) | Not Specified | Unspecified Outcome Score at Unclear Interval | High LPR was associated with Poor Outcome | Not Specified | High LPR is Associated with Poor Outcome |
| Goodman et al [25] | *Mixed Peri-Lesion and Healthy Tissue*  ***Lactate and Glucose***  (Unclear Interval) | Various ICP Therapies; DC | Unspecified Outcome Scale at Unclear Interval | High median LGR (mean 6.7 versus 2.7)(p=0.004) and High Lactate (mean 0.85 umol/mL versus 0.68 umol/mL) (p=0.04) were Associated with Poor Outcome | Not Specified | Low Glucose and High Lactate is Associated with Poor Outcome |
| Gopinath et al [27] | *Unclear Location*  ***Glutamate***  (Unclear Interval) | Not Specified | GOS at Unclear Interval | High Glutamate is associated to Poor Outcome (p<0.001) | Not Specified | High Glutamate is Associated with Poor Outcome |
| Gupta et al [29] | *Peri-Lesional*  ***Glucose, Lactate, Pyruvate, Glutamate, Glycerol***  (Hourly Measure for 3 to 5 days) | DC after Failure of Medical ICP Therapy | Dichotomized GOS at 3 Months (Good = 4 or 5) | The number of low CMD Glucose episodes was associated with Poor Outcome (p<0.0026) | Not Specified | Low Glucose Episodes Are Associated with Poor Outcome at 3 Months |
| Gupta et al [28] | *Unclear Location*  ***Glycerol, Lactate, Pyruvate, LPR***  (Unclear Interval) | ICP/CPP Directed Therapy | GOS at unspecified interval | Glycerol levels (within 1^st^ 2 days) were associated with outcome (mean 220.86 umol/L versus 191.76 umol/L)  LPR was associated with outcome (mean 80.16 versus 45.77) (p=0.000) | Not Specified | Glycerol and LPR are associated with outcome |
| Hejcl et al [34] | *Unclear Location*  ***Glucose, Glycerol, LPR***  (Unclear Interval) | Not Specified | Unspecified Outcome Scale at 6 Months | High Glycerol (mean 215 umol/L versus 62.07 umol/L) and LPR (mean 87.97 versus 37.36) were associated with Poor Outcome at 6 Months (p<0.05) | Not Specified | High Glycerol and LPR are Associated with Poor Outcome at 6 Months |
| Hutchinson et al [38] | *Unclear Location*  ***Glucose, Lactate, LPR***  (Unclear Interval) | Not Specified | Unspecified Outcome Scale at Discharge from Hospital | Low Glucose (mean 1.39 mmol/L versus 1.8 mmol/L) and High Lactate (mean 5.1 mmol/L versus 4.38 mmol/L)/LPR (mean 35.5 versus 27.9) were associated with Poor Outcome  High Glutamate Levels and Spikes were associated with Poor Outcome | Not Specified | Low Glucose and High Lactate/LPR/Glutamate are Associated with Poor Outcome at Discharge from Hospital |
| Igarashi et al [42] | *Unclear Location*  ***Glucose, Lactate, Pyruvate, Glycerol, Glutamate***  (Hourly Measure for 24 hours) | Not Specified | Unspecified Dichotomization of GOS at unclear interval | High Lactate (mean 87.2 mmol/L versus 39.3 mmol/L) (p=0.026), Glycerol (mean 1288 umol/L versus 142 umol/L) (p<0.001) and LPR (mean 80.8 versus 31.9) (p=0.002) at 24 hours post injury were associated with Poor Outcome | Not Specified | High Lactate/Glycerol/LPR at 24 hours are associated with Poor Outcome |
| Johnston et al [44] | *Normal Brain*  ***Glucose, Lactate, Pyruvate, LPR, Glycerol***  (Measure q30 min; unclear how correlated to outcome) | CPP Directed Therapy with augment to >70 mm Hg | Unspecified Outcome Scale at Unclear Interval | Baseline LPR was higher (mean 36 versus 17) in the poor outcome group  No clear association with individual measured CMD substrates and outcome | Not Specified | High LPR is associated with Poor Outcome |
| Karathanou et al [45] | *Unclear Location*  ***Glycerol and LPR***  (Unclear Interval) | Not Specified | Dichotomized GOS at 6 Months (Good = 4 or 5; Poor = 3 or less) | High Glycerol and LPR were associated with Poor Outcome | Not Specified | High Glycerol/LPR are associated with Poor Outcome at 6 Months |
| Koura et al [47] | *Unclear Location*  ***Glutamate***  (Unclear Interval for 5 days) | Not Specified | Unspecified Outcome Scale at Unclear Interval | Mean Glutamate over first 5 days is associated with outcome (p=0.0234) | Not Specified | High Glutamate (within the first 5 days) is associated with Poor Outcome |
| Kurtz et al [48] | *Unclear Location*  ***Glucose, Lactate, LPR***  (Hourly Measure) | ICP Therapies; Hypothermia | Mortality in Hospital | Mean Brain/Serum Glucose ratio <0.12 is associated with hospital mortality | Not Specified | Low Brain Glucose is associated with in-hospital morality |
| Li et al [51] | *Mixed Peri-Lesion and Healthy Tissue*  ***Glycerol***  (Hourly Measure) | Various ICP Therapies | Mortality and “Poor” Outcome at unspecified interval | High Glycerol levels were associated with poor outcome and mortality (p<0.05) | Not Specified | High Glycerol is associated with poor outcome |
| Marcoux et al [53] | *Healthy Tissue*  ***Glucose, Lactate, Pyruvate, Glutamate***  (Hourly Measure) | Not Specified | 6 Month GOSE | Strong correlation percentage of between time with elevated LPR (>40) and poor outcome at 6 months (p<0.01) | Not Specified | High LPR is associated with Poor Outcome at 6 Months |
| Mazzeo et al [54] | *Healthy Tissue*  ***Glucose, Lactate, Pyruvate, Glutamate***  (Unclear Intervals for 5 days) | ICP Directed Therapies | 3 Month GOS | High Lactate (p<0.01) and High LGR (p<0.01) were associated with Poor Outcome at 3 Months | Not Specified | High Lactate/LGR is associated with Poor Outcome at 3 Months |
| Mellergard et al [55] | *Unclear Location*  ***Glutamate, Glycerol, lactate, Pyruvate, LPR***  (q2 Hour Measure grouped into 6 hour collections) | Not Specified | Mortality at 6 Months | Higher mean Glycerol, Glutamate, Lactate, LPR associated with Mortality at 6 months | Not Specified | High Glycerol, Glutamate, Lactate, and LPR are associated with Mortality at 6 Months |
| Nordstrom et al [62] | *Mixed Peri-Lesion and Healthy Tissue*  ***Glucose, Lactate, Glutamate, Glycerol***  (Hourly Measure) | Lund Therapy | Mortality at 6 Months | Total hours with LPR >30 and Pyruvate <70 umol/L was associated with mortality at 6 months in all injury patterns  Total hours LPR>30 and Pyruvate >70 umol/L was only associated with mortality in SDH group | Not Specified | Elevated LPR and Low Pyruvate is associated with mortality at 6 months |
| Oddo et al [64] | *Mixed Peri-Lesion and Healthy Tissue*  ***Glucose, Lactate, Pyruvate***  (Hourly Measure) | ICP Therapies; Intensive; Pentobarbital in some; Insulin therapy via IV (goal: 4.4 to 6.7 mmol/L) | Mortality at unspecified interval | Low CMD Glucose (mean 0.46 mmol/L versus 1.04 mmol/L) was associated with mortality | Not Specified | Low Glucose is associated with mortality |
| Olivecrona et al [65] | *Bilateral Placement (Peri-Lesion and Healthy Tissue)*  ***Glucose, Lactate, Pyruvate, LPR***  (q2 hour Measure) | Lund Therapy for ICP  RCT of Epoprostenol (n=24) vs. Placebo (n=24) | Mortality at 3 Months | LPR was higher in the non-survivors (mean 86.2 versus 62.5)  Epoprostenol has not impact on LPR or patient outcome | No Complications | Elevated LPR is associated with mortality at 3 Months |
| Omerhodzic et al [66] | *Mixed Peri-Lesion and Healthy Tissue*  ***Glucose, Lactate, Pyruvate, LPR, Glycerol***  (Unclear Interval) | Not Specified | Unspecified Outcome Scale | Low glucose associated with outcome at 1 year  No association between LPR/glycerol and outcome | Not Specified | Low Glucose is associated with poor outcome at 1 year |
| Paraforou et al [69] | *Peri-Lesional*  ***Glucose, Glycerol, Pyruvate, Lactate***  (q2 hour Measure) | ICP Directed Therapy | 6 Month GOS | High Glycerol (>72 mmol/L) and LPR (>37) were associated with Poor Outcome at 6 Months (p=0.007 and 0.000 respectively)  Glucose was not associated with outcome | Not Specified | High Glycerol/LPR are associated with Poor Outcome at 6 Months |
| Peerdeman et al [70] | *Healthy Tissue*  ***Glucose, Lactate, Pyruvate, Glycerol***  (Unclear Interval) | ICP/CPP Directed Therapy; some barbiturates and hypothermia | Dichotomized GOS at 6 Months (Good = 4 or 5; Poor = 3 or less) | The total Glycerol measured within the first 24 hours was associated with outcome (mean 4440 umol/hr versus 991 umol/hr) (p=0.044)  Peak Glycerol levels >150 umol/L were associated with mortality  Sensitivity/Specificity in Outcome prediction is 60%/100% respectively | Not Specified | Total Glycerol in the first 24 hours is associated with outcome at 6 Months (high glycerol = poor outcome) |
| Petzold et al [71] | *Mixed Peri-Lesion and Healthy Tissue*  ***Lactate, Pyruvate, LPR***  (Hourly Measure) | Not Specified | GOS at 3 and 6 Months | The percentage of patient hours with LPR > 25 (p=0.0003) was associated with poor outcome | Not Specified | LPR >25 is associated with poor outcome at 3 and 6 months |
| Reinert et al [73] | *Unclear Location*  ***Glucose, Lactate***  (Hourly Measure) | Not Specified | Dichotomized GOS at 12 Months (Good = 4 or 5; Poor = 1 to 3) | High Glucose (mean 2045 umol/L versus 1273 umol/L) was associated with good outcome (p<0.0001)  High Lactate (mean 2437 umol/L versus 1590 umol/L) was associated with poor outcome (p<0.0001) | Not Specified | Glucose and Lactate are associated with outcome at 12 Months |
| Reinert et al [74] | *Mixed Peri-Lesion and Healthy Tissue*  ***Potassium, Lactate, Glutamate***  (q30 min Measure) | Not Specified | Dichotomized GOS at 3 Months (Good = 4 or 5; Poor = 1 to 3) | Low mean potassium (<1.8 mmol/L) was associated with good outcome (p<0.0001) | Not Specified | Low Potassium is associated with a good outcome at 3 months |
| Richards et al [75] | *Healthy Tissue*  ***Glutamate***  (Hourly Measure) | ICP Therapies | GOS at 3 Months | High Glutamate levels (at 12h and 24h post injury) were associated with poor outcome | Not Specified | Glutamate at 12h and 24h post injury correlate to outcome at 3 months |
| Robertson et al [76] | *Unclear Location*  ***Glutamate***  (q30 min Measure) | Not Specified | Unspecified Outcome Scale at Unclear Interval | High Mean Glutamate and Aspartate levels were associated with mortality (p=0.04) | Not Specified | High Glutamate levels are associated with mortality |
| Sanchez-Porras et al [81] | *Unclear Location*  ***Glucose, Lactate, Pyruvate, Glutamate***  (Hourly Measure) | Local Protocol; Individual differences | Dichotomized GOS at 6 Months (Alive = 2 to 5; Dead = 1) | Increases in Lactate (p=0.016) and Glutamate (p=0.021) correlated negatively to outcome | Not Specified | Increased Lactate and Glutamate are associated with poor outcome |
| Sanchez et al [80] | *Unclear Location*  ***Glucose, Lactate, Pyruvate, LPR***  (Unclear Interval) | Not Specified | Dichotomized GOS at “discharge” (Good = 4 or 5; Poor = 1 to 3) | High Glucose, Lactate and LPR were associated with poor outcome  Low pyruvate was associated with poor outcome | Not Specified | High Glucose, Lactate, and LPR (with low pyruvate) are associated with poor outcome at discharge |
| Singla et al [86] | *Unclear Location*  ***LPR***  (Unclear Interval) | Not Specified | GOS at 6 Months | High LPR (mean 80.16 versus 45.77) was associated with poor outcome | Not Specified | High LPR is associated with poor outcome at 6 months |
| Stein et al [88] | *Healthy Tissue*  ***Glucose, Lactate, Pyruvate, LPR***  (Hourly Measure for 10 days) | Various ICP Directed Therapies | Dichotomized GOSE at 6 Months (Good = 7 or higher; Poor = below 7) | Development of “metabolic crisis” and duration of elevated LPR (>25) and low glucose (<0.8 mmol/L) was associated with outcome  (p=0.011)  Elevated Glutamate levels were associated with poor outcome | Not Specified | Both the development and duration of metabolic crisis (LPR >25 and glucose <0.8 mmol/L) were associated with outcome at 6 months |
| Stiefel et al [91] | *Unclear Location*  ***Lactate, Pyruvate, LPR***  (Hourly Measure for 3 days) | Not Specified | Unspecified Outcome Scale at Unclear Interval | Development and Duration of “Impaired” Multi-modal Monitoring (MMM)(CPP/PbtO_2_/NIRS) in combination with Metabolic derangements (LPR >25) (as per CMD on an hourly basis) correlated to poor outcome  Abnormal CMD or MMM alone failed to display a statistically significant correlation to outcome | Not Specified | MMM in combination with CMD assessment of metabolic disturbance correlates to outcome |
| Timofeev et al [93] | *Mixed Peri-lesion and Healthy Tissue*  ***Glucose, Lactate, Pyruvate, Glutamate, Glycerol, LPR***  (Hourly Measure) | Not Specified | Dichotomized GOS at 6 Months (Good = 4 or 5; Poor = 1 to 3) | *72hr median values correlated to outcome:*  Low Lactate (p=0.033), LPR (<25)(p=0.026), glycerol (p=0.014), and Glutamate (p=0.046) levels correlated to good outcome  *Median values over entire monitoring period correlated to outcome:*  Glucose (p=0.024) and LPR (p=0.016) | Not Specified | Low Lactate, LPR (<25), glycerol and Glutamate over the first 72hrs are associated with Good Outcome at 6 Months  Median Glucose and LPR for entire monitoring period is associated with outcome at 6 months |
| Vespa et al [99] | *Healthy Tissue*  ***Glucose, Lactate***  (Hourly Measure) | Not Specified | GOSE at 6 Months | Mean hourly glucose values trend (over the first 10 days) was associated with outcome – with low hourly means linked to poor outcome (p<0.0001)  Similar trend seen for lactate and LPR (p<0.0001) | No Complications | The trend in mean hourly CMD glucose, lactate, and LPR (within the first 10 days of injury) are associated with outcome at 6 months |
| Wang et al [103] | *Unclear Location*  ***Glucose, Glutamate, Glycerol, Lactate***  (Hourly Measure) | Post DC, CPP targeted (>70 mm Hg) and PbtO_2_ targeted (>20 mm Hg) therapy | Dichotomized GOS at unclear interval (Good = 4 or 5; Poor = 1 to 3) | Low LPR at 24h and 72h post-injury was associated with good outcome  No association was found between Glucose/Glycerol/Glutamate/Lactate and outcome | Not Specified | Low LPR at 24h and 72h post injury is correlated to good outcome in DC patients |
| Yokobori et al [105] | *Lesional (Penumbra)*  ***Glucose, Glutamate, Glycerol, Lactate, Pyruvate, LPR***  (Hourly Measure) | Standard ICP/CPP directed therapy | Dichotomized GOS (Good 4 or 5; Poor = 1 to 3) at unclear interval | Low glucose (mean 13.8 mmol/L versus 19.9 mmol/L) and elevated LPR (mean 151.1 versus 35.5)were associated with poor outcome  **NOTE: The mean glucose numbers were quoted within a table as this high. We suspect the units were supposed to be mg/L and not mmol/L*  High glutamate (mean 71.1 umol/L versus 23.4 umol/L) and glycerol (mean 695.0 umol/L versus 217.0 umol/L) levels were seen in the poor outcome group | Not Specified | Low Glucose and Elevated LPR/Glutamate/Glycerol were associated with poor outcome |
| Zauner et al [107] | *Healthy Tissue*  ***Glucose, Lactate***  (Unclear Interval) | Not Specified | Unspecified Outcome Scale at unclear interval | Low CMD glucose (mean 639 umol/L versus 808 umol/L) and High Lactate (mean 1642 umol/L versus 1001 umol/L) were associated with poor outcome | Not Specified | Low Glucose and High Lactate are associated with poor outcome |
| Zauner et al [108] | *Healthy Tissue*  ***Lactate, Glucose***  (q30 min for 4 days) | Not Specified | Trichotomized GOS at 3 to 6 Months | Low CMD Glucose and High Lactate was associated with poor outcome | Not specified | Low Glucose and High Lactate are associated with poor outcome |
| **Nil Association Studies** | | | | | | |
| Alessandri et al [3] | *Unclear Location*  ***Sodium, Glutamate***  (Unclear Interval) | Not Specified | Unspecified Outcome Scale at unclear interval | High Sodium was not associated with outcome  Glutamate was not associated with outcome | Not Specified | Sodium and Glutamate are not associated with outcome |
| Chen et al [16] | *Unclear Location*  ***Lactate, Pyruvate, LPR, Glucose***  (Unclear Interval) | Not Specified | Unspecified Outcome Scale at Unclear Interval | Mean hourly LPR and Glucose were not associated with poor outcome | Not Specified | CMD LPR and Glucose were not associated with outcome |
| Nelson et al [60] | *Bilateral Location (one in penumbra; one in healthy)*  ***Glucose, Lactate, Pyruvate, LPR, Glutamate***  (Hourly Collection) | Standard ICP/CPP Therapy | Trichotomized GOS (Dead = 1; Poor = 2 or 3; Good = 4 or 5) at unclear interval | No association between CMD measures and GOS | Not Specified | Glucose, Lactate, LPR and Glutamate are not associated with outcome |
| Nelson et al [61] | *Mixed Peri-lesion and Healthy*  ***Glucose, Lactate, Pyruvate, Glycerol***  (Hourly Measures) | ICP and CPP directed Therapy; some with DC, barbiturates, hypothermia | Dichotomized GOS at 3 to 6 Months (Good = 4 or 5; Poor = 1 to 3) | No significant association between any CMD measures and outcome | Not Specified | Glucose, Lactate, Pyruvate, LPR and Glycerol do not correlate with outcome at 3 to 6 months |
| Papanikolaou et al [68] | *Unclear Location*  ***Lactate, Pyruvate, LPR, Glycerol***  (Unclear Interval) | Not Specified | Unspecified Outcome Scale at Unclear Interval | Not clear association between CMD measures and outcome | Not Specified | Lactate, Pyruvate, LPR and Glycerol do not correlate with outcome |
| Thelin et al [92] | *Lesional*  ***Lactate, Pyruvate, Glucose***  (Hourly Measure) | ICP/CPP Directed Therapy | Dichotomized GOSE at 6 Months (Good = 7 or 8; Poor = 1 to 6) | No association between brain CMD measures and patient outcome | Not Specified | Glucose, Lactate and Pyruvate are not associated with 6 month outcome |

TBI = traumatic brain injury, GOS = Glasgow outcome scale, GOSE = Glasgow outcome scale extended, CMD = cerebral microdialysis, RCT = randomized control trial, ICP = intracranial pressure, CPP = cerebral perfusion pressure, NAA = N-acetyl acetate, CSF = cerebrospinal fluid, LPR = lactate:pyruvate ratio, CBF = cerebral blood flow, rCBF = regional cerebral blood flow, SjvO2 = jugular venous oxygen saturation, MABP = mean arterial blood pressure, PbtO2 = partial pressure of oxygen in brain tissue, Mx = autoregulation, PRx = pressure reactivity monitoring, TCD = transcranial Doppler, PET = positron emission tomography, FDG = fluorodeoxyglucose, OEF = oxygen extraction fraction, NIRS = near infrared spectroscopy, MMM = multi-modal monitoring, DC = decompressive craniectomy, mmol = milli-molar, mm Hg = millimeters of mercury, L = liter, umol = micromolar.
