# Supplementary material for: A systematic review of cerebral microdialysis and outcomes in TBI: relationships to patient functional outcome, neurophysiologic measures, and tissue outcome
Source: Acta Neurochir (Wien). 2017 Oct 7;159(12):2245–73. doi: 10.1007/s00701-017-3338-2 (PMC5686263; doi:10.1007/s00701-017-3338-2)
Supplement: Supplementary file 6 — (DOC 131 kb) [file 701_2017_3338_MOESM6_ESM.doc]

Appendix F: Neurophysiologic Measure Studies - CMD Measures and Physiologic Associations

| **Reference** | **Catheter Location and**  **Measured CMD Analytes of Interest** | **Interventional Therapies Applied During Measurement** | **Physiologic Parameter Measured** | **CMD Analytes and**  **Physiologic Outcome** | **Complications to CMD** | **Conclusions** |
| --- | --- | --- | --- | --- | --- | --- |
| ***ICP/CPP Positive Association Studies*** | | | | | | |
| Adamides et al [1] | *Mixed Peri-Lesion and Healthy Tissue*  ***Glucose, Lactate, Pyruvate, Glutamate, Glycerol***  (Hourly Measure) | Standard ICP/CPP Directed Therapy | ICP/CPP | Labile ICP was associated with labile LPR, Lactate, and Glutamate levels. Episodes of elevated ICP/low CPP were associated with elevations in Lactate, LPR, and Glutamate which occurred 2 to 3 hours prior.  Absolute Lactate elevation, peak lactate, baseline lactate, and baseline LPR/glycerol were associated with elevated ICP/low CPP (p<0.05) | Not Specified | Absolute Lactate elevation, peak lactate, baseline lactate, and baseline LPR/glycerol were associated with elevated ICP/low CPP |
| Belli et al [7] | *Peri-Lesional Tissue*  ***Lactate, Pyruvate, LPR, Glutamate***  (q12 Hour Measure) | Standard ICP/CPP Directed Therapy; Varied end-tier ICP therapies (DC, etc.) | ICP | Elevated LPR, glutamate, and glycerol were correlated to increased ICP (p<0.001)  LPR >25 and Glycerol >100 umol/L were associated with abnormal ICP (p<0.001) | No Complications | Elevated LPR, glutamate, and glycerol were correlated to increased ICP |
| Bolcha et al [10] | *Unclear Location*  ***Glucose, Glycerol, LPR***  (Unclear Interval) | Not Specified | ICP and PbtO_2_ | *ICP:* High ICP was associated with high LPR and Glycerol  *PbtO_2_:* High LPR correlated with low PbtO_2_ | Not Specified | High LPR and Glycerol are associated with high ICP. High LPR is associated with low PbtO_2_. |
| Bullock et al [12] | *Mixed Peri-Lesion and Healthy Tissue*  ***Glutamate***  (q30 min Measure for 4 days) | Standard ICP Therapy; “Few” with barbiturate infusions | ICP/CPP | High Glutamate (>20 umol/L) was associated with elevated ICP/low CPP (p=0.0104)  Glutamate was linearly correlated to the severity of secondary ischemic episodes (defined by ischemia score; PaO_2_, MABP, Xe CT, pupil response, CPP) (p=0.025) | 3 catheters malfunctioned requiring replacement | High Glutamate is associated with elevated ICP |
|  |  |  |  |  |  |  |
| Clausen et al [17] | *Healthy Tissue*  ***Glycerol***  (q30 min Measure) | Various ICP Therapies | CPP and PbtO_2_ | *PbtO_2_:* Glycerol levels negatively correlated with PbtO_2_ (p<0.001)  *CPP:* Glycerol levels negatively correlated with CPP (p<0.05) | Not Specified | High Glycerol levels are associated with low PbtO_2_ and low CPP. |
| Clausen et al [18] | *Healthy Tissue*  ***Lactate***  (q30 min Measure) | Various ICP Therapies; Barbiturates; Hypothermia | CPP and PbtO_2_ | *CPP:* High lactate observed in episodes of low CPP (mean 1027 umol/L), with the largest mean change in lactate correlating significantly (p<0.05)  *PbtO_2_:* High lactate was associated with low PbtO_2_. (p<0.05) | Not Specified | High Lactate is with low CPP and PbtO_2_. |
| Goodman et al [26] | *Healthy Tissue*  ***Glutamate***  (q 30min Measure) | Various ICP Therapies (including Barbiturates) | ICP | As Glutamate decreases (with progressive sedation) so does ICP | Not Specified | Glutamate Levels are Associated with ICP |
| Goodman et al [25] | *Mixed Peri-Lesion and Healthy Tissue*  ***Lactate and Glucose***  (Unclear Interval) | Various ICP Therapies; DC | ICP, SjVO_2_, PbtO_2_ | *ICP:* Lactate increases and Glucose decreases as ICP continuously increases  *SjvO_2_:* Elevated Lactate (median increase >0.322 umol/L) was associated to decrease in SjvO_2_ (p=0.002). The change in Glucose was related to the severity of SjVO_2_ decrease (p=0.032)  *PbtO_2_:* Increased in Lactate did not correlate to local changes in PbtO_2_. | Not Specified | Increases in Lactate and Glucose were associated with an increase in ICP and decrease in SjvO_2_ |
| Gupta et al [28] | *Unclear Location*  ***Glycerol, Lactate, Pyruvate, LPR***  (Unclear Interval) | ICP/CPP Directed Therapy | ICP | LPR and CPP displayed a negative correlation (p=0.029) | Not Specified | Elevations in LPR are associated with a decrease in CPP |
| Hejcl et al [34] | *Unclear Location*  ***Glucose, Glycerol, LPR***  (Unclear Interval) | Not Specified | ICP/CPP, PbtO_2_ | *ICP/CPP:* High LPR associated with elevated ICP (p<0.05), and low CPP (p<0.05)  High Glycerol associated with elevated ICP (p<0.05)  *PbtO_2_:* High LPR was associated with low PbtO_2_ (p<0.05) | Not Specified | High LPR is associated with high ICP and low CPP/PbtO_2_. High Glycerol is associated with high ICP |
| Koura et al [47] | *Unclear Location*  ***Glutamate***  (Unclear Interval for 5 days) | Not Specified | ICP | Elevated Glutamate was associated with elevated ICP | Not Specified | High Glutamate is associated with ICP elevations |
| Kurtz et al [48] | *Unclear Location*  ***Glucose, Lactate, LPR***  (Hourly Measure) | ICP Therapies; Hypothermia | CPP and PbtO_2_ | *CPP:* Low Glucose is associated with low CPP  *PbtO_2_*: High LPR and Low Glucose is associated with low PbtO_2_ | Not Specified | Low glucose is associated with low CPP/PbtO_2_. High LPR is associated with low PbtO_2_ |
| Li et al [51] | *Mixed Peri-Lesion and Healthy Tissue*  ***Glycerol***  (Hourly Measure) | Various ICP Therapies | ICP/CPP and CBF (Laser Doppler Flowmetry) | *ICP/CPP:* High Glycerol was associated with decreased CPP and increased ICP  *CBF:* High Glycerol was associated with reduced CBF | Not Specified | High Glycerol is associated with high ICP and low CPP/CBF |
| Meixensberger et al [56] | *Peri-Lesional*  ***Glucose, Lactate, Pyruvate, LPR,*** ***Glutamate***  (q1-2 Hour Measures) | Variable Surgical and Medical Treatments for ICP | ICP and PbtO_2_ | *ICP:* Glutamate was only associated with severe spikes in ICP in some cases  *PbtO_2_:* Elevated LPR (>25) was associated with low PbtO_2_ (p<0.001) | CMD catheter failure in 5 | High Glutamate is sometimes associated with ICP spikes. Elevated LPR is associated with low PbtO_2_. |
| Nelson et al [61] | *Mixed Peri-lesion and Healthy*  ***Glucose, Lactate, Pyruvate, Glycerol***  (Hourly Measures) | ICP and CPP directed Therapy; some with DC, barbiturates, hypothermia | ICP/CPP | Strong correlation between ICP/CPP and all CMD measures (ie. Low glucose, high lactate/LPR/glycerol seen in episodes of high ICP/low CPP) | Not Specified | Low glucose, high lactate/LPR/glycerol seen in episodes of high ICP/low CPP |
| Nordstrom et al [62] | *Mixed Peri-Lesion and Healthy Tissue*  ***Glucose, Lactate, Glutamate, Glycerol***  (Hourly Measure) | Lund Therapy | CPP | Low CPP (<20 mmHg) corresponded to increase in LPR | Not Specified | LPR increases when CPP drops dramatically |
| Papanikolaou et al [68] | *Unclear Location*  ***Lactate, Pyruvate, LPR, Glycerol***  (Unclear Interval) | Not Specified | ICP | Elevated Glycerol predicted ICP increase | Not Specified | Lactate, Pyruvate, LPR and Glycerol do not correlate with outcome |
| Paraforou et al [69] | *Peri-Lesional*  ***Glucose, Glycerol, Pyruvate, Lactate***  (q2 hour Measure) | ICP Directed Therapy | ICP | High LPR and Glycerol occurred during times of sustained ICP elevations | Not Specified | High LPR and Glycerol may be seen in circumstances of elevated ICP |
| Richards et al [75] | *Healthy Tissue*  ***Glutamate***  (Hourly Measure) | ICP Therapies | ICP | Mean glutamate levels positively correlated to ICP (p<0.05)  No significant correlation between hourly glutamate and ICP/CPP/MABP/CVP | Not Specified | Glutamate at 12h and 24h post injury correlate to outcome at 3 months |
| Salci et al [79] | *Unclear Location*  ***Lactate, Pyruvate, LPR***  (Unclear Interval) | Non-specific therapies directed at ICP/CPP | ICP and Compliance | High LPR correlates with High ICP and Poor Compliance (p<0.0001) | Not Specified | High LPR is associated to increased ICP and poor compliance |
| Sarrafzadeh et al [82] | *Healthy Tissue*  ***Glucose, Lactate, Glycerol, Glutamate***  (Hourly Measure) | Not Specified | ICP, SjvO_2_ and PbtO_2_ | *ICP:* High ICP (>20 mm Hg) was associated with a 4-5 fold increase in Glutamate  *SjvO_2_:* Low SjvO_2_ was associated with elevated Glutamate, Lactate, and Glycerol  *PbtO_2_:* Low PbtO_2_ was associated with elevated Glutamate, Lactate, and Glycerol | Not Specified | High Glutamate is associated with high ICP and low SjvO_2_/PbtO_2_. High Lactate and Glycerol is associated with low SjvO_2_/PbtO_2_ |
| Singla et al [86] | *Unclear Location*  ***LPR***  (Unclear Interval) | Not Specified | ICP | LPR and Glycerol levels increase as CPP decreased | Not Specified | LPR and Glycerol levels increase as ICP increases |
| Stahl et al [87] | *Mixed Uni- and Bilateral in Peri-Lesion and Healthy Tissue*  ***Lactate, Pyruvate, Glutamate, Glycerol***  (q30-60 min Measure) | Varied Failed ICP Therapies | ICP | As ICP progressively increases towards herniation, Glycerol/Glutamate/LPR increase and Glucose decreases | Not Specified | As ICP increases to herniation, Glycerol/Glutamate/LPR increase, while Glucose decreases |
| Stein et al [89] | *Unclear Location*  ***Glucose, Lactate, Pyruvate***  (Unclear Interval for 7 days) | Unclear ICU protocols | ICP | Metabolic crisis (LPR>40) was associated with elevated ICP | Not Specified | Elevated LPR may be associated with sustained ICP elevations |
| Stiefel et al [91] | *Unclear Location*  ***Lactate, Pyruvate, LPR***  (Hourly Measure for 3 days) | Not Specified | CPP | Impaired Metabolism (LPR>25) was found in associated with abnormal MMM (varied techniques; CPP, NIRS, PbtO_2_) | Not Specified | Elevated LPR (>25) may be found in episodes of abnormal MMM |
| Timofeev et al [93] | *Mixed Peri-lesion and Healthy Tissue*  ***Glucose, Lactate, Pyruvate, Glutamate, Glycerol, LPR***  (Hourly Measure) | Not Specified | ICP and PRx | *ICP:* Glutamate, Glycerol and LPR were found to elevated in those with high mean ICP values  *PRx:* Glutamate, Glycerol and LPR were found to be elevated in those with positive PRx values | Not Specified | Elevated Glutamate, Glycerol and LPR may be seen in those with high ICP and abnormal PRx |
| Timofeev et al [94] | *Unclear Location*  ***Lactate, Pyruvate, LPR***  (Unclear Interval) | Not Specified | ICP/CPP, PRx, PbtO_2_ | *ICP/CPP:* Increased LPR was seen with an increase in ICP and decrease in CPP  *PRx:* Increased LPR was associated with abnormal PRx  *PbtO_2_:* Increased LPR was seen in the setting of low PbtO_2_ | Not Specified | Increased LPR may be seen in settings of High ICP, low CPP/PbtO_2_, and abnormal PRx |
| Vespa et al [98] | *Unclear Location*  ***Glutamate***  (Hourly Measure; 9 days) | Standard ICP/CPP Directed Therapy; Barbiturates intermittently | ICP/CPP | Glutamate elevations (>20 uM) were seen in episodes of decreased CPP (p<0.001) and increased ICP at various time points during admission | No complications | Glutamate elevations (>20 uM) may be seen in episodes of decreased CPP and elevated ICP |
| ***PbtO2/SjvO2 Positive Association Studies*** | | | | | | |
| Chan et al [15] | *Unclear Location*  ***Glucose, Lactate, Glutamate, Glycerol***  (Unclear Interval) | Not Specified | SjvO_2_ | Rapid increase in Glutamate, Glycerol and Lactate were ween with SjvO_2_ levels below 40 to 45%  Glucose decreased with SjvO_2_ below 42% | Not Specified | Increase in Glutamate/Glycerol/Lactate and Decrease in Glucose can be seen with a decrease in SjvO_2_ to 40 to 45% |
| Figaji et al [21] | *Unclear Location*  ***Lactate, LPR, glycerol***  (Unclear Interval) | Not Specified | PbtO_2_ | LPR increased with low PbtO_2_ | Not Specified | High LPR is associated with low PbO_2_ |
| Menzel et al [57] | *Unclear Location*  ***Glucose, Lactate***  (q30 min Measure) | Varied therapies directed toward ICP  *Group 1 (n=12):* FiO_2_ directed to PaO_2_ of 100 to 150 mm Hg  *Group 2 (n=12):* Sequential increase in FiO_2_ to 60% and then 100% | PbtO_2_ | Lactate decreased during increase in PaO_2_ and PbtO_2_  Glucose displayed no relationship | Not Specified | Lactate decreases with increased PbtO_2_ |
| Menzel et al [58] | *Unclear Location*  ***Glucose, Lactate***  (q30 min Measure) | Unclear therapies;  N=14 with hyperoxia therapy – sequential increase in FiO_2_ up to 100% | PbtO_2_ | During baseline measures there was no correlation between lactate and glucose with PbtO_2_  During hyperoxia Lactate decreased with a corresponding increase in PbtO_2_.  Glucose showed no clear trend with PbtO_2_ druring hyperoxia. | Not Specified | Lactate may decrease with increasing PbtO_2_ |
| Nortje et al [63] | *Unclear Location*  ***Glucose, Lactate, Pyruvate, Glutamate, Glycerol***  (q20 min Measure) | Standardized Protocol  Hyperoxia Therapy: Unclear | PbtO_2_ and ^15^O PET | *PbtO_2_:* LPR decreased as PbtO_2_ increased during hyperoxia. Not significant changes were note for lactate, pyruvate, glucose, glutamate and glycerol in isolation.  *PET:* ^15^O PET update focally was seen with hyperoxia, but this did not correlate to LPR changes. | Not Specified | LPR may decrease with increasing PbtO_2_ |
| Purins et al [72] | *Unclear Location*  ***Glucose, Lactate, Pyruvate, Glutamate, Glycerol***  (Hourly Measure) | ICP/CPP directed therapies; variable treatments | PbtO_2_ | Glutamate increases significantly with PbtO_2_ below 5 mm Hg (p<0.05)  Glycerol increases significantly with PbtO_2_ below 5 mm Hg (p<0.05)  LPR increase with PbtO_2_ below 5 mm Hg (p<0.01)  No significant correlation between glucose and PbtO_2_ | Not Specified | LPR, Glutamate and Glycerol increase as PbtO_2_ drops below 5 mm Hg. Glucose doesn’t respond to decrease PbtO_2_ levels. |
| Robertson et al [76] | *Unclear Location*  ***Glutamate***  (q30 min Measure) | Not Specified | PbtO_2_ and CBF (TCD) | High Glutamate/Aspartate levels were negatively correlated to diffuse brain injury, PbtO_2_, and CBF (TCD) | Not Specified | High Glutamate levels are associated with mortality |
| Sarrafzadeh et al [83] | *Unclear Location*  ***Glucose, Lactate, Pyruvate, LPR, Glutamate***  (Hourly Measure) | Not Specified | PbtO_2_ | As PbtO_2_ dropped to 10-15 mm Hg (for 5 min), glutamate increased (p=0.006)  As PbtO_2_ drops below 10 mm Hg (5 min), Glutamate (p<0.001), Lactate (p=0.001), and LPR (p=0.088) increased | Not Specified | Increases in Glutamate, Lactate and LPR can be seen with decreasing PbtO_2_ |
| Sarrafzadeh et al [84] | *Unclear Location*  ***Glucose, Lactate, LPR, Glutamate***  (Hourly Measure) | Not Specified | PbtO_2_ | Glucose dropped significantly prior to PbtO_2_ decreasing to 10 mm Hg.  Glutamate displayed no relationship to PbtO_2_ | Not Specified | Decreased glucose may predict PbtO_2_ defined hypoxia. Glutamate does not seem to correlate. |
| Timofeev et al [85] | *Mixed Peri-Lesion and Healthy Tissue*  ***Glucose, Lactate, Pyruvate, LPR, Glycerol, Glutamate***  (Hourly Measure) | Standardized protocol; individual patients not all same | PbtO_2_ | Strong negative correlation between LPR (p<0.001) and lactate (p<0.001) and PbtO_2_ | Not Specified | LPR may correlate to PbtO_2_ |
| Valdaka et al. [96] | *Unclear Location*  ***Glucose, Lactate,***  ***Glutamate***  (q30 min Measure) | Non-specific treatment | PbtO_2_ | Lactate, Glutamate, LPR and potassium increase significantly as PbtO_2_ approaches zero. | Not Specified | Limited. Significant derrangements seen during death. |
| Vilalta et al [101] | *Healthy Tissue*  ***Glucose, Lactate, Pryuvate, Glutamate***  (Hourly Measure) | ICP/CPP therapy; no very clear  Hyperoxia Therapy: obtain baseline, then increase FiO_2_ to 100% | PbtO_2_ | During hyperoxia increases in PbtO_2_ (mean increased from 26.9 mm Hg to 97.7 mm Hg) occur in concert with increases in glucose (p=0.037) and decreases in LPR (p=0.005), lactate (p<0.001) and pyruvate (p=0.002) | Not Specified | Increased glucose with decreases in LPR/Lactate/Pyruvate may be seen with increases in PbtO_2_ |
| ***Autoregulation - Positive Association Studies*** | | | | | | |
| Asgari et al [4] | *Unclear Location*  ***Lactate, Pyruvate, LPR***  (Unclear Interval) | Unclear Therapy | MOCAIP ICP Waveform based Autoregulation Measurements | Occasional LPR increases seen in circumstances of autoregulation dysfunction | Not Specified | LPR may be increased in situation with abnormal autoregulation |
| Asgari et al [5] | *Unclear Location*  ***Lactate, Pyruvate, LPR***  (Unclear Interval) | Not Specified | PMTM ICP Waveform Analysis for Vascoconstriction/Vasodilatation | Some association between LPR increase and vasoconstriction | Not Specified | Increased LPR may correlated to vasoconstriction in some cases |
| Yokobori et al [105] | *Peri-Lesional*  ***Glucose, Glutamate, Glycerol, Lactate, Pyruvate, LPR***  (Hourly Measure) | Standard ICP/CPP directed therapy | PRx | There was a positive correlation between improvement in PRx and CMD glucose levels  There was a negative correlation between improved PRx and LPR | Not Specified | LPR and Glucose changes may correlate to PRx based changes |
| ***Imaging Based Positive Association Studies*** | | | | | | |
| Bouzat et al [11] | *Healthy Tissue*  ***Lactate, Pyruvate, LPR, Glucose***  (Unclear Interval) | ICP/CPP Directed Therapy; no specifics | CTP assessment of CBF during episodes of brain hypoxemia (PbtO_2_ <20 mm Hg for 5 min) or “Abnormal” CMD measures (Glucose <1 mmol/L and LPR >40) | Low Glucose and elevated LPR were associated with a reduction in rCBF (p<0.05) | Not Specified | Low Glucose and elevate LPR correlated to a reduction in rCBF as per CTP |
| Hutchinson et al [39] | *Unclear Location*  ***Glucose, Lactate, Pyruvate, Glutamate***  (q20 min Measure) | Standard Protocols; no specifics | H_2_^15^O PET (with ROI around probe location) | Positive correlation between OEF and LPR (p=0.002) | Not Specified | LPR and OEF seem to be positively correlated |
| Hutchinson et al [40] | *Unclear Location*  ***Glucose, Lactate, Pyruvate, Glutamate***  (q30 min Measure) | Standard Protocol; no further details | FDG PET (ROI around probe location) | Lactate and Pyruvate displayed a positive linear correlation to CMRglc (p<0.0001)  Weak positive correlation between CMRglc and Glutamate  No relationship between CMRglc and LPR or Glucose was seen | Not Specified | Lactate, Pyruvate and Glutamate displayed a positive correlation to CMRglc |
| Reinert et al [74] | *Mixed Peri-Lesion and Healthy Tissue*  ***Potassium, Lactate, Glutamate***  (q30 min Measure) | Not Specified | Xe CT and ICP | Negative correlation between potassium and CBF (p=0.019)  Positive correlation between mean potassium and ICP >30 mm Hg (p<0.0001) | Not Specified | Potassium has a negative correlation to Xe CT CBF |
| Sala et al [78] | *Unclear Location*  ***Glucose, Lactate, Pyruvate, Glutamate***  (Hourly Measure) | Standard ICP/CPP Protocol | CTP at 24 and 48 hours post injury  ICP | *CTP:* Glycolytic Lactate elevations (normal PbtO_2_) were associated with normal/supranormal perfusion. Hypoxic lactate elevations (PbtO_2_ < 20 mm Hg for 5 min) were associated with global oligemia.  *ICP:* Glycolytic lactate elevations were not associated with ICP elevations | Not Specified | Hypoxic Lactate elevations are associated with global oligemia |
| Vespa et al [97] | *Unclear Location*  ***Glucose, Lactate, Pyruvate, Glutamate, Glycerol***  (Hourly Measure) | Standard ICP/CPP Directed Therapy | FDG PET and C^15^O, O^15^O, H_2_^15^O PET (ROI around probe location) | LPR was negatively correlated to CMRO_2_ (p<0.001)  No other correlations detected | Not Specified | LPR displays a negative correlation to CMRO_2_ |
| Vespa et al [99] | *Healthy Tissue*  ***Glucose, Lactate***  (Hourly Measure) | None Mentioned | FDG PET (ROI around probe location) and Xe CT | Mean daily CMD glucose = 0.05 to 3.2 mmol/L  CMD glucose value of 0.2 mmol/L (or lower) corresponded to an increase in FDG signal on PET  CMD glucose positively correlated with Xe CT CBF measurements (p<0.001) | No Complications | Glucose is positively correlated to FDG PET signal and Xe CT CBF |
| Zauner et al [109] | *Unclear Location*  ***Glutamate***  (Unclear Interval) | Not Specified | Xe CT | Elevations in glutamate corresponded positively to reductions in CBF | Not Specified | Elevations in glutamate may correspond to reductions in CBF as assessed by Xe CT |
| ***Negative Association Studies*** | | | | | | |
| Alessandri et al [3] | *Unclear Location*  ***Sodium, Glutamate***  (Unclear Interval) | Not Specified | ICP | No correlation between Sodium/Glutamate with ICP was identified | Not Specified | Sodium and Glutamate levels do not correlate with ICP |
| Chamoun et al [13] | *Unclear Location*  ***Glutamate***  (Hourly Measure) | Various ICP Therapies; Some DC patients | ICP/PbtO_2_/SjvO_2_ | No correlation between glutamate and MABP/ICP/PbtO_2_/SjvO_2_ | Not Specified | Glutamate does not correlate to MABP/ICP/PbtO_2_/SjvO_2_ |
| Johnston et al [44] | *Healthy Brain*  ***Glucose, Lactate, Pyruvate, LPR, Glycerol***  (Measure q30 min; unclear how correlated to outcome) | CPP Directed Therapy with augment to >70 mm Hg | CPP and PbtO_2_ | No association between CMD measures and CPP  Unclear association between CMD measures and PbtO_2_ | Not Specified | CMD does not correlate with CPP or PbtO_2_ changes |
| Nelson et al [60] | *Bilateral Location (one in penumbra; one in healthy)*  ***Glucose, Lactate, Pyruvate, LPR, Glutamate***  (Hourly Collection) | Standard ICP/CPP Therapy | ICP/CPP | No common patterns of CMD measures were seen in relation to ICP/CPP | Not Specified | CMD measure do not correlated to ICP/CPP changes |
| Peerdeman et al [70] | *Healthy Tissue*  ***Glucose, Lactate, Pyruvate, Glycerol***  (Unclear Interval) | ICP/CPP Directed Therapy; some barbiturates and hypothermia | ICP/CPP | No association between low CPP and increased glycerol  No association between elevated ICP and glycerol  No association between elevated temperature and glycerol | Not Specified | Glycerol elevations are not associated with ICP/CPP changes |
| Petzold et al [71] | *Mixed Peri-Lesion and Healthy Tissue*  ***Lactate, Pyruvate, LPR***  (Hourly Measure) | Not Specified | ICP | Unclear relationship between Lactate, Pyruvate and LPR to ICP | Not Specified | Questionable relationship between Lactate, Pyruvate and LPR to ICP |
| Sanchez-Porras et al [81] | *Unclear Location*  ***Glucose, Lactate, Pyruvate, Glutamate***  (Hourly Measure) | Local Protocol; Individual differences | Long PRx (using 20 min averages of MAP and ICP data) | No correlation was seen between CMD measures and Long PRx | Not Specified | Glucose, Lactate, Pyruvate, Glutamate do not correlate with Long PRx defined autoregulation |
| Vespa et al [100] | *Mixed Peri-Lesion and Healthy Tissue*  ***Lactate, Pyruvate, LPR***  (Hourly Measure; mean duration 120 hours) | Not Specified | CPP | LPR changes occur independent of CPP changes. | Not Specified | LPR changes occur independent of CPP changes. |

TBI = traumatic brain injury, GOS = Glasgow outcome scale, GOSE = Glasgow outcome scale extended, CMD = cerebral microdialysis, RCT = randomized control trial, ICP = intracranial pressure, CPP = cerebral perfusion pressure, NAA = N-acetyl acetate, CSF = cerebrospinal fluid, LPR = lactate:pyruvate ratio, CBF = cerebral blood flow, rCBF = regional cerebral blood flow, SjvO2 = jugular venous oxygen saturation, MABP = mean arterial blood pressure, PbtO2 = partial pressure of oxygen in brain tissue, Mx = autoregulation, PRx = pressure reactivity monitoring, TCD = transcranial Doppler, PET = positron emission tomography, FDG = fluorodeoxyglucose, OEF = oxygen extraction fraction, ROI = region of interest, CMRglc = cerebral metabolic rate of glucose consumption, CMRO_2_ = cerebral metabolic rate of oxygen consumption, Xe CT = xenon enhanced computed tomography, CTP = compute tomographic perfusion imaging, NIRS = near infrared spectroscopy, MMM = multi-modal monitoring, DC = decompressive craniectomy, mmol = milli-molar, mm Hg = millimeters of mercury, L = liter, umol = micromolar.
