# Supplementary material for: A systematic review of cerebral microdialysis and outcomes in TBI: relationships to patient functional outcome, neurophysiologic measures, and tissue outcome
Source: Acta Neurochir (Wien). 2017 Oct 7;159(12):2245–73. doi: 10.1007/s00701-017-3338-2 (PMC5686263; doi:10.1007/s00701-017-3338-2)
Supplement: Supplementary file 7 — (DOC 34 kb) [file 701_2017_3338_MOESM7_ESM.doc]

Appendix G: Tissue Outcome Studies - CMD Measures and Tissue Outcome

| **Reference** | **Catheter Location and**  **Measured CMD Analytes of Interest** | **Interventional Therapies Applied During Measurement** | **Technique to Measure Tissue Outcome** | **CMD Analytes and**  **Tissue Outcome** | **Complications to CMD** | **Conclusions** |
| --- | --- | --- | --- | --- | --- | --- |
| **Positive Association Studies** | | | | | | |
| Filippou et al [23] | *Unclear Location*  Lactate, Pyruvate, LPR, Glucose  (Unclear Interval) | None Mentioned | NAA, Cho, Cr based MRS and ADC Evaluation | No signs of ADC defined ischemia were seen  LPR displayed a negative correlation with NAA (p=0.037) and Cr (p<0.001)  Glucose displayed a positive correlation with Cho (p=0.007) and NAA/Cho (p=0.028) | None Mentioned | LPR is negatively correlated with NAA and Cr; Glucose is positively correlated with Cho and NAA/Cho |
| Marcoux et al [53] | *Healthy Tissue*  Glucose, Lactate, Pyruvate, Glutamate  (Hourly Measure) | None Mentioned | MRI Volumetric Assessment of Frontal Lobe Atrophy at 6 Months Post-Injury | Degree and duration of LPR elevation negatively correlated to MRI volumetric based frontal lobe atrophy at 6 months (p<0.01)  Mean LPR in 1^st^ 96 hours is associated with degree of atrophy (p<0.01)  LPR >40 during the initial 72 and 96 hours correlated to atrophy (p<0.01) | None Mentioned | The Degree and Duration of LPR Elevation correlates to frontal lobe atrophy at 6 months |
| **Nil Association Studies** | | | | | | |
| Filippou et al [22] | *Unclear Location*  Lactate, Pyruvate, LPR  (Unclear Interval during 1^st^ 7 days) | None Mentioned | NAA, Cr based MRS and ADC Evaluation | No association between elevated LPR and NAA/Cr | None Mentioned | LPR is not associated with MRS based NAA/Cr changes |
| Stein et al [89] | *Unclear Location*  Lactate, Pyruvate, LPR, Glucose  (Unclear Interval) | None Mentioned | MRI based ADC assessment for ischemia | No correlation between ADC and CMD substrates were identified | None Mentioned | CMD Defined Metabolic Crisis is not associated with MRI based signs of ischemia |

CMD = cerebral microdialysis, LPR = lactate:pyruvate ratio, NAA = N-acetyl acetate, Cr = Creatinine, Cho = Choline, MRS = Magnetic Resonance Spectroscopy, MRI = Magnetic Resonance Imaging, ADC = Apparent Diffusion Coefficient, GOSE = Glasgow Outcome Score Extended, mmol = milimolar, L = liter.
