# Supplementary material for: A systematic review of cerebral microdialysis and outcomes in TBI: relationships to patient functional outcome, neurophysiologic measures, and tissue outcome
Source: Acta Neurochir (Wien). 2017 Oct 7;159(12):2245–73. doi: 10.1007/s00701-017-3338-2 (PMC5686263; doi:10.1007/s00701-017-3338-2)
Supplement: Supplementary file 8 — (DOC 27 kb) [file 701_2017_3338_MOESM8_ESM.doc]

**Appendix H: “Nil” Association Studies - Summary**

1. *Functional Outcome Studies*

Six studies documented the absence of an association between any of the CMD measures and patient functional outcome. [3,16,60,61,68,92] The majority found a lack of significance association between glucose, glutamate and lactate levels when compared with GOS at 3 to 6 months. The total number of patients in these studies was 214, a minority of the total number of patients across all studies in this review.

1. *Neuro-physiologic Measure Studies*

No association between ICP and/or CPP and common CMD measures was described in 7 studies, [3,13,44,60,70,71,100] totaling 281 patients. No association between ICP and/or CPP was found for glutamate in 3 studies, [3,13,60] sodium in 1 study, 32] glycerol in 2 studies, [44,70] LPR in 2 studies, [71,100] and all measures in 2 studies. [44,60] In general, the studies reporting absence of association tended to include small numbers of patients, with only 1 study having more than 30 patients. [13]

No association between CMD glutamate and SjvO2 was described in 1 study with 135 patients. [13]

Six studies, totaling 315 patients, found no relationship between one or more common CMD measures and PbtO_2_ values. One study found no relationship between mean lactate levels and PbtO_2_. [25] No association was found between glutamate and PbtO_2_ in 2 studies. [13,64] Two studies found no relationship between mean glucose and PbtO_2_. [21,58] One study found no relationship between all CMD measures and PbtO_2_. [44]

One study on 29 patients reported no association between a different technique of autoregulation measurement (“long PRx”) and all common CMD measures. [81]

Two studies found no association between some common CMD measures and imaging based physiologic measures, totaling 28 patients [40,63] utilizing ^15^O and FDG PET techniques.

1. *Tissue Outcome Studies*

Two studies were identified as “nil association” given their inability to find any association between CMD measures and tissue outcome. One study was a meeting abstract detailing the use of MRS and MRI ADC assessment of tissue outcome in the acute/subacute phase post-injury, at an unspecified interval. [22] There was no association found between CMD measured lactate, pyruvate or LPR and MRS or ADC assessment. The second study was a meeting abstract describing MRI based ADC assessment for ischemia/infarction in the acute/subacute period post-injury, at an unspecified interval. [89] There was no association between CMD measures and MRI based ischemia. Metabolic crisis, defined as an elevated LPR (above undisclosed threshold) was found to have no correlation to late ADC in the region of the CMD catheter.
